# Characterization of early transcriptional responses to cadmium in the root and leaf of Cd-resistant *Salix matsudana* Koidz

**DOI:** 10.1186/s12864-015-1923-4

**Published:** 2015-09-17

**Authors:** Jingli Yang, Kun Li, Wei Zheng, Haizhen Zhang, Xudong Cao, Yunxiang Lan, Chuanping Yang, Chenghao Li

**Affiliations:** State Key Laboratory of Forest Genetics and Tree Breeding, Northeast Forestry University, 26 Hexing Road, Harbin, 150040 China; School of Forestry, Northeast Forestry University, 26 Hexing Road, Harbin, 150040 China; Research Center on Life Sciences and Environmental Sciences, Harbin University of Commerce, Harbin, 150076 China

**Keywords:** *Salix matsudana* Koidz, Cadmium, Brassinosteroids, Transcriptome

## Abstract

**Background:**

*Salix matsudana* Koidz. is a fast growing tree species. It has a high cadmium (Cd) tolerance capacity, making it potentially suitable for phytoremediation. Presently, transcriptomic and physiological Cd response mechanisms are poorly understood. Transcriptomic analysis in early response to high (50 μM) Cd levels was investigated in leaf and root of Cd-resistant *S. matsudana* Koidz..

**Results:**

Analysis of the response profiles demonstrate the existence of a complex transcriptional network in the root and leaf when exposed to Cd. The main response in the root involved up-regulation of genes associated with defence response via callose deposition in the cell wall and cell wall thickening. In the leaf, transcripts related to biotic stress signalling and secondary metabolism were activated. Additionally, many lignin and brassinosteroids synthesis pathway genes were induced mainly in the leaf, indicating that gene response to Cd was tissue-specific. The Cd transcriptome results were consistent with observed physiological changes.

**Conclusion:**

The sub-localization, transcriptional network, and physiological regulation demonstrate the tissue-specific manner of Cd response, and provide a novel insight into in early response of tree species to Cd exposure.

**Electronic supplementary material:**

The online version of this article (doi:10.1186/s12864-015-1923-4) contains supplementary material, which is available to authorized users.

## Background

Cadmium (Cd) is one of the most toxic nonessential elements for plants. Accumulation of Cd into the food chain via crop root absorption from soil environment is a threaten to human health. This makes it necessary to clean-up Cd contaminated soils. A promising remediation technologies is phytoremediation, the use of plants to clean up polluted soils [1]. Suitable plants for phytoremediation should possess multiple traits such as fast growth, high biomass production, easy harvest and high accumulation of a range of heavy metals in their harvestable parts [2]. Willow is a fast growing, productive and deeply rooted tree, well adapted to temperate region climatic conditions, tolerated temporary water-logging [3]. Recent research has shown that willow has considerable potential for the phytoremediation of heavy metal contaminated land and has the capacity to accumulate elevated levels of Cd in aboveground biomass compartments [4, 5]. Among various clones, *Salix matsudana* Koidz. is a Chinese willow species belonging to the Salicaceae family, and is native to northeastern China. It has been reported that some clones of *S. matsudana* have high heavy metal tolerance [6].

The molecular mechanisms underlying the adaptation to metals in herbaceous model plants are well studied. Phytochelatins (PCs) and glutathione (GSH) can bind Cd iron forming cd-ligands and were transported to vacuoles [7]. It is reported that metallothioneins (MTs) and PCs work cooperatively to protect *Arabidopsis thaliana* from Cu and Cd toxicity [8]. Cd can enter the plant symplast through ZIP transporters [9]. Additionally, heavy metal ATPase (HMA) plays a key role in cellular metal efflux [10–12]. Generally, Cd stress finally activates antioxidative enzymes responsive. However, trees could have evolved also distinct mechanisms to cope with elevated metal concentrations. Their elucidation requires broad scale transcriptional, proteomic and metabolic approaches. The high-throughput next generation sequencing (NGS) technology such as RNA-seq and Digital Gene Expression (DGE) is an efficient method to illustrate the mechanisms at molecular level. Compared with traditional gene cloning, NGS technology has characteristics with high efficiency, fast run times and high accuracy [13, 14]. Among the different types of NGS technology, the Illumina Hiseq system has been widely used [15, 16], owing to its high throughput, accuracy, and low costs. Up to date, only a few study on transcriptome sequensing are available for willow [17–19]. However, the transcriptional responses of Salix to Cd contamination remain unknown. This study investigated the early transcriptional response to a high Cd (50 μM) treatment for 10 h in the root and leaves of *S. matsudana* plants by *de novo* transcriptome analysis. The identified molecular and physiological mechanisms provide a novel insight into Cd tolerance in tree species, and play a foundational role in phytoremediation investigations.

## Methods

### Plant material and cadmium exposure

Open-pollinated mature seeds of *S. matsudana* Koidz. var. matsudana were collected during late May from 60-year-old trees grown on the campus of Northeast Forestry University, Harbin, China. Seeds were stored and sterilized as described by Yang *et al.* [20], and inoculated on 1/2 MS medium [21] supplemented with 150 μM Cd (CdCl_2_), without plant growth regulators (PGRs), for 1 month (Additional file 1: Figure S1a). Cd resistant seedlings were then cultured in the same medium without Cd at successive 4-week intervals. When regenerated seedlings reached a height of 5 cm, the materials were cultured in 1/2 MS medium containing 50 μM Cd. Before this, we treated *S. matsudana* with different concentration of Cd, 50 μM is the highest concentration that neither affect the phenotype nor the growth, thus chose this concentration. To determine mechanisms in which cadmium accumulation altered molecular functioning in tissues at an early stage (10 h), three independent individual of leaves and roots were respectively collected and pooled into one sample to be sequenced using Illumina paired-end sequencing technology for *de novo* transcriptomic analysis. Non-treated materials were used as control.

Media used in experiments were adjusted to pH 5.8 before adding 0.8 % Plant agar (Duchefa, The Netherland), and then sterilized by autoclaving at 1.1 kg cm^−2^ (121 °C) for 15 min. The cultures were incubated under a 16 h light/8 h dark photoperiod at 25 °C under illumination at 45 μmol m^−2^ s^−1^ with cool white fluorescent lights.

### cDNA library preparation and sequencing

Total RNA was extracted using the hexadecyltrimethylammonium bromide (CTAB) method [22], and digested with RNase-free DNase I (Promega, Madison, USA) at 37 °C for 30 min. The concentration and integrity of RNA samples for transcriptome analysis were evaluated using a NanoPhotometer (GmbH, Munich, Germany). The quality and quantified of extracted RNA was assessed using an Agilent 2100 Bioanalyzer (Agilent Technologies, Mississauga, Canada) with an RNA integration number (RIN) of 8, which is an algorithm for assigning integrity values to RNA measurements. For transcriptome analysis, the cDNA library was prepared using the TruSeq Sample Preparation Kit (Illumina, San Diego, CA, USA) following the manufacturer’s instructions. The samples were listed as following (Table 1) and clustered in flow cells to construct the cDNA library to be sequenced by the Illumina HiSeqTM 2000.

**Table 1:** Description of libraries for the sequence running

Sample name	Insert size (nt)	Quality (ng/μl)	Concentration (nM)	Standard QPCR concentration (nM)	Total volume (μl)
Con_leaf	211	8.98	40.63	84.92	2.47
Cd_leaf	203	9.66	44.73	74.15	2.83
Con_root	217	8.72	38.8	71.07	2.95
Cd_root	234	8.58	36.32	66.51	3.16

The sequencing data from this study were deposited in the NCBI Sequence Read Archive (SRA, http://www.ncbi.nlm.nih.gov/Traces/sra/) under accession number SRR1167984.

### De novo assembly, annotation and MapMan analysis

Clean reads were obtained by deleting empty reads, adaptor sequences, and low-quality sequences. The clean reads were assembled into contigs using Trinity [23], and the contigs were connected using Unigene. For functional annotation, the de novo transcriptomic target sequences were used to blast against the Phytozome Populus trichocarpa version 2.2 transcript database. BlastX alignment (*E*-value <10^−5^) was performed between unigenes and the following protein databases: NCBI non-redundant protein (nr) (http://www.ncbi.nlm.nih.gov/), Swiss-Prot (http://www.expasy.org/), KEGG (http://www.genome.jp/kegg), and COG (http://www.ncbi.nlm.nih.gov/COG). The highest sequence similarity to a gene in the NCBI Nr database was annotated to the Unigene. Gene Ontology (GO) annotations for the Unigenes were determined by Blast2GO [24], and WEGO software [25] was used to analyze GO functional classifications for all Unigenes.

To categorize differentially expressed genes according to their biological functions, the closest Arabidopsis homolog (*Arabidopsis* gene identifiers, AGIs) of a *S. matsudana* gene was determined was determined by a translated nucleotide BLAST (BLASTx) of the coding sequence of the best *P. trichocarpa* hit against the Arabidopsis protein sequence data set. Annotations were taken from the latest release of The Arabidopsis Information Resource genome, TAIR10.0. Differentially expressed genes were submitted to MapMan v3.5.1 (http://gabi.rzpd.de/projects/MapMan/) [26] for coexpression analysis as described by Wei *et al.* [27].

### Evaluation of the libraries

Rigorous algorithms were developed to identify differentially expressed genes between two samples. The correlation of the detected count numbers between parallel libraries was assessed statistically by calculating the Pearson correlation. *P* ≤0.01, FDR ≤0.1, and the absolute value of log2Ratio ≥1 were used as threshold to assess the significance of gene expression difference. For pathway enrichment analysis, we mapped all differentially expressed genes to terms in KEGG database and looked for significantly enriched KEGG terms compared to the genome background.

### Quantitative real time polymerase chain reaction (qRT-PCR) analysis

Total RNA was extracted from Cd-treated and non-treated leaf and root tissue of *S. matsudana* plants using the CTAB method [22], respectively. First-strand cDNA synthesized with 0.5 μg purified RNA was reverse-transcribed with a Reverse Transcriptase kit (TaKaRa Biotech, Dalian, China). PCR reaction was performed in a volume of 20 μl, containing 10 μl of SYBR premix ExTaq (TaKaRa Biotech, Dalian,China), 0.5 μM of forward and reverse primers, and 2 μl cDNA template (equivalent to 0.05 μg of total RNA). Thermal cycling conditions was performed as following: 10 s at 95 °C followed by 40 cycles of 5 s at 95 °C, 30 s at 60 °C, and 1 s at 78 °C for plate reading. The primer sequences of *β*-tubulin and 18 randomly selected genes of leaves are given in the Additional file 2: Table S1.

### Chlorophyll content of leaf tissues

Fresh leaf tissue (1 g) of plant was exposed to 50 μM Cd for 10 h and collected to determine chlorophyll content. These samples were soaked in 80 %-acetone solution (5 mL) and kept in the dark at 4 °C for 24 h. The supernatant was collected for analysis in a spectrophotometer (722S, Shanghai Lengguang Technology Co., Ltd, China). Optical densities were recorded at 663 nm and 645 nm to determine the concentrations (μg g^−1^ fresh mass) of chlorophyll a and chlorophyll b, respectively, and of total chlorophyll (a + b) in each sample. Non-treated plant was used as control. Each experimental unit was consisted of 10 explants with three replicates.

### Analysis of cadmium content

Roots, stems and leaves of *S. matsudana* treated with 50 μM Cd for 10 h or 1 month were dried at 80 °C until to a constant weight. Samples (0.1–0.2 g) were then dissolved in a mixture of concentrated HNO_3_–HClO_4_ (5:1, v/v) and heated at 160 °C for 5 h. Following cooling, the extract was filtered, and the beaker washed with 3 ml 6 M HCl. Following treatment, aliquots were obtained to analyse residual Cd content in the tissues using an atomic absorption spectrophotometer (Atomic Absorption Spectrometer, TAS-986). Non-treated samples were used as the control.

### TEM observation

Leaves and root segments (approximately 2–3 mm long) of plant treated with 50 μM Cd for 1 month were collected, and fixed in 2.5 % glutaraldehyde for 2 days. They were then vacuumed at room temperature, washed with PBS (pH = 6.8) buffer for 15 min three times, and then fixed with OsO_4_ for 3–4 h. Samples were dehydrated through an ethanol series, and embedded in Spurr’s epoxy resin. Ultrathin sections were obtained using a Power Tome XL ultramicrotome (RMC-Boeckeler Instruments, Tucson, AZ, USA), collected on copper-supported grids, and observed using an H-7650 transmission electron microscope (TEM, Hitachi High-Technologies, Tokyo, Japan).

### Callose detection

Plant materials were cultured in 1/2 MS medium supplemented with 50 μM Cd for 2 weeks. Root and leaf callose were detected using aniline blue (Sigma-Aldrich, St. Louis, MO, USA) and observed under a light microscope (Olympus SZ61, Tokyo, Japan).

### Histochemical staining of cadmium

Cadmium localization was performed as described by He *et al.* [28]. Briefly, sections of root and leaf of 50 μM Cd treated for 10 h and non-treated plants were soaked in a staining solution containing 30 mg diphenylthiocarbazone dissolved in 60 mL acetone, 20 mL water, and 100 mL glacial acetic acid for 1 h, and then rinsed in deionized water. The prepared section or samples were photographed under a light microscope (Olympus SZ61, Tokyo, Japan).

### Brassinosteroids inhibition assay

For plant growth investigation, seedlings cultured in 1/2 MS medium with the addition of 50 μM Cd via cutting propagation were subjected to the following treatment conditions: (1) Non-treatment. (2) 50 μM Cd. (3) 10 nM brassinazole (Brz, a specific inhibitor of brassinosteroid (BR) biosynthesis) (4) 50 μM Cd +10 nM brassinazole. (5) 50 μM Cd + 20 nM Brz. (6) 50 μM Cd + 50 nM Brz; all treatments were performed for 2 weeks. Non-treated seedlings were used as a control.

### Detection of ROS and determination of superoxide dismutase, catalase, and ascorbate peroxidase activity

The quantity of O_2_^−^ and H_2_O_2_ in leaves and roots of plant treated with 50 μm Cd for 10 h was monitored by incubation with 2 mM nitroblue tetrazolium (NBT; N6876, Sigma, St. Louis, MO, USA) in 20 mM phosphate buffer (pH 6.1) containing 10 mM NaN_3_, or in 3,3′ diaminobenzidine (DAB; D5637, Sigma–Aldrich) (pH 3.8), respectively. Chlorophyll was destained by boiling in alcohol (95 %, v/v) for 1 h as described by Xu *et al.* [29].

Leaf tissues (0.5 g) from Cd-treated (50 μM Cd for 10 h) and non-treated plants were ground to a powder in liquid nitrogen. Superoxide dismutase (SOD), catalase (CAT), and ascorbate peroxidase (APX) activities were measured as described previously [30].

### Statistical analysis

All experiments were repeated independently for at least three replicates. Statistical analyses were carried out using SPSS 16.0 for Windows (SPSS Inc., Chicago, IL, USA). Data were compared using Student’s *t* test. Differences were considered to be significant if *P* <0.05.

## Results

### Plant growth

For Cd-resisitant *S. matsudana* selection, only five seedlings showed relatively vigorous growth from approximately 2000 seeds screened, but their following growth was delayed. After successive culture on medium without Cd, the seedlings were screened on medium supplemented with 50 μM Cd for 1 month. Under Cd treatment conditions, the number of root was higher in Cd resistant lines (Additional file 1: Figure S1b, right) than in non-resistant lines (Additional file 1: Figure S1b, left), and leaves of Cd resistant lines remained green (Additional file 1: Figure S1b, right), whereas leaves of control check (CK) plants withered and yellowed (Additional file 1: Figure S1b, left). One of the Cd-resistant plant lines was selected for the following analyses.

### Chlorophyll content and cadmium accumulation

Chlorophyll a, b, and a + b content was reduced in the Cd-treated plant compared with non-treated plants (Additional file 1: Figure S2a). The Cd distribution pattern was then investigated when exposed to Cd for 1 month (Additional file 1: Figure S2b), the highest Cd concentration was found in the roots, and it was lower in the stem and leaf. The Cd concentrations in the analysed tissues were 852.77 (root), 197.96 (stem), and 65.98 (leaf) μg g^−1^ dry weight, thus confirming hyperaccumulation. Cd was not observed in control plant tissues (Additional file 1: Figure S2b).

**Fig. 1:**
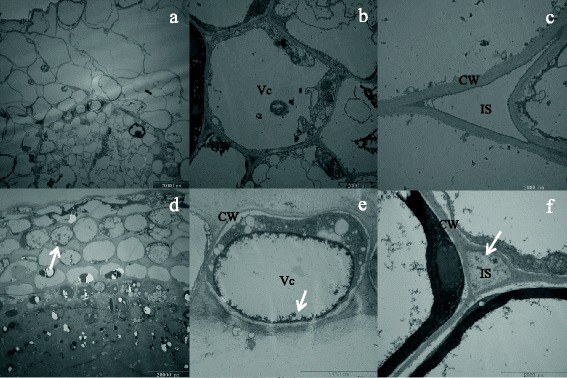
Transmission electron microscope observation of *Salix matsudana* Koidz. root cell. **a** Cross section of a non-treated plant root. **b** Single cell of a non-treated plant root. **c** Intercellular space of a non-treated plant root. **d** Cross section of a Cd-treated plant root. **e** Single cell of a Cd-treated plant root. **f** Intercellular space of a Cd-treated plant root. *CW* Cell wall, *Vc* Vacuole, *IS* Intercellular space. The *arrows* stand for Cd deposits

TEM results revealed that the cortical cells of non-treated roots were not organized (Fig. 1a). No Cd was detected in the cells (Fig. 1b) or intercellular space (Fig. 1c). The shape of root cells changed following 50 μM Cd treatment for 1 month, illustrating that Cd damaged plant development. The cortical cells were smaller and neater, with observable cell wall thickening (Fig. 1d). There were some Cd deposits in the vacuole (Fig. 1e) and intercellular space (Fig. 1f). But no considerably differences in leaves between treated and non-treated samples (data not shown).

### Cadmium localization in cortical root cells

**Fig. 2:**
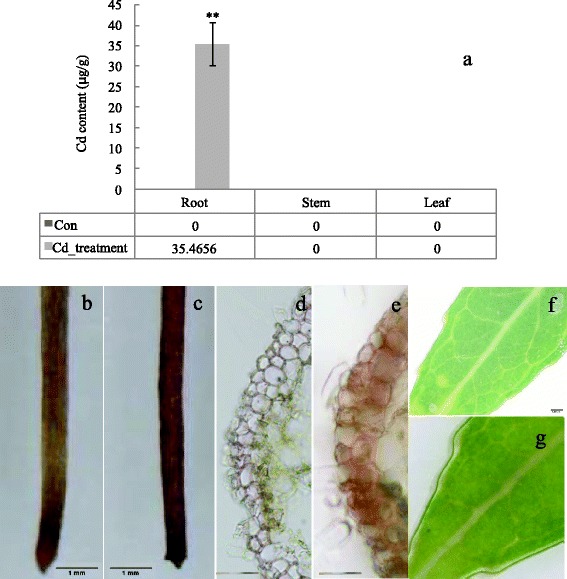
Cadmium concentration in tissues, and localization in the root of *S. matsudana* Koidz.. **a** Cd concentration following 50 μM Cd treatment for 10 h. **b** Non- and (**c**) Cd-treated roots stained with diphenylthiocarbazone solution. **d** Cross section of non-treated root stained with diphenylthiocarbazone solution. **e** Cross section of Cd-treated root stained with diphenylthiocarbazone solution. **f** Non- and (**g**) Cd-treated leaves stained with diphenylthiocarbazone solution. Bar = 1 mm

Cd concentration was measured in various tissues in the early response to Cd (for 10 h). The results revealed that only a small amount of Cd (35 μg/g. FW) had accumulated in the root at this point (Fig. 2a), indicating that root-to-shoot translocation does not exist at this early stage. Histochemical staining for Cd was conducted to investigate cellular localization of Cd in tissues at this stage (Fig. 2b–e). A large difference was detected between non- and Cd-treated (50 μM) roots. Greater dark-brown staining was observed in the Cd-treated root (Fig. 2c) compared with the non-treated sample (Fig. 2b). From the root cross section, it was observed that cadmium-dithizone staining was mainly detected in cortical cells (Fig. 2e); staining was not detected in the control plant (Fig. 2d). It is noteworthy that there was no difference between control (Fig. 2f) and Cd-treated plant leaves (Fig. 2g).

### Illumina sequencing and de novo assembly

Because of the absence of reference genomic sequences, *de novo* assembly was applied to construct transcripts from RNA-seq reads.

Following cleaning of data, high quality reads were obtained for control and Cd-treated leaf and root samples (Additional file 2: Table S2). Following assembling, high quality reads were assembled to contigs, and further assembled to unigenes (Additional file 2: Table S3).

### Functional annotation

A sequence similarity search was conducted against the NCBI Nr database. In the Nr classifications, 66.2 and 78.3 % of mapped unigenes showed significant homology (Additional file 1: Figure S3a and b). Numerous unigenes (85.2 %) showed homology with poplar (*Populus trichocarpa*; Additional file 1: Figure S3c).

Unigenes were searched against the COG database for functional prediction and classification. Total 29,894 sequences were assigned to COG classifications (Additional file 2: Table S4, Additional file 1: Figure S3d). Among the 24 COG categories, the cluster for general function prediction only (17.1 %) was the largest group, followed by transcription (9.5 %).

Based on Nr annotations, 316,118 unigenes were assigned gene ontology (GO) terms, there were 37,909 functional terms. Among the three major GO classifications, annotated unigenes assigned to cellular biological processes made up the largest set (156,617, 49.5 %), followed by cellular components (113,307, 35.8 %), and molecular function (46,194, 14.6 %) (Additional file 2: Table S5, Additional file 1: Figure S3e). The functionally assigned unigenes covered a comprehensive range of GO categories.

### Comparison of leaf and root in response to cadmium using MapMan

After exposure to 50 μM Cd for 10 h, 912 genes were determined to be up-regulated and 669 down-regulated in the leaf, while 448 genes were up-regulated and 459 genes down-regulated in the root. To gain further insight into the coexpression of functional gene response to Cd, the unique AGIs were selected in *S. matsudana*. In all, 447 homologs were identified in the leaf (Additional file 2: Table S6) and 145 homologs (Additional file 2: Table S7) in the root, these were further analysed in MapMan.

**Fig. 3:**
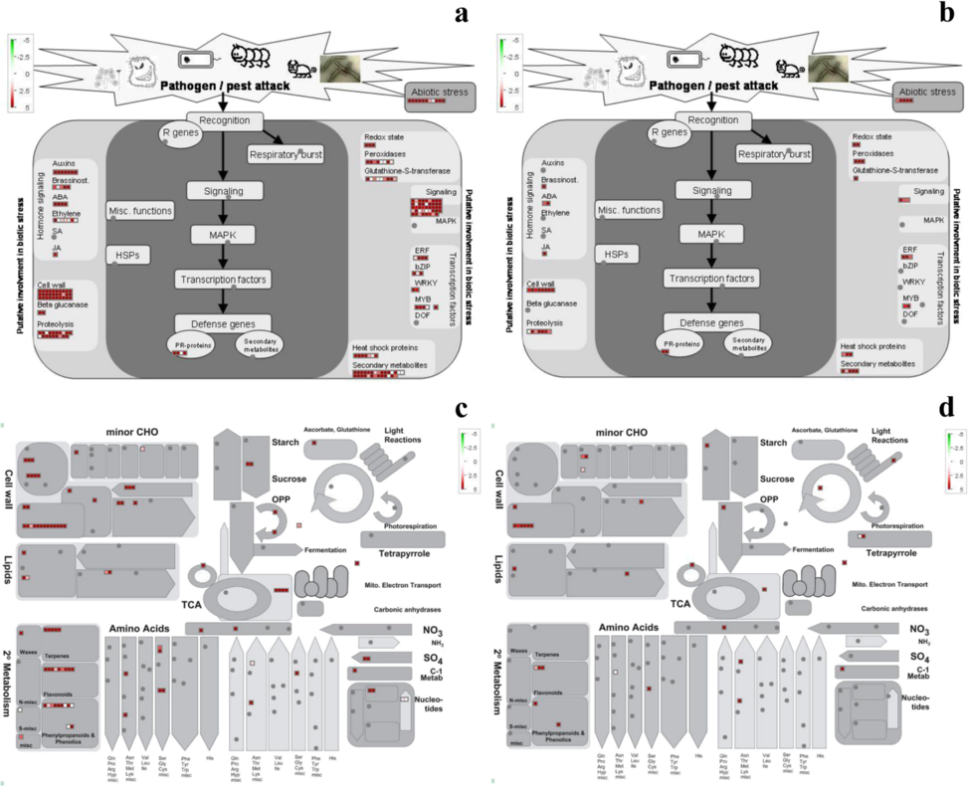
MapMan analysis of genes involved in stress in leaf (**a**) and root (**b**), and metabolism in leaf (**c**) and root (**d**). *Red* represents up-regulation and *green* down-regulation

MapMan analysis revealed that almost all genes identified as being up-regulated under biotic stress and involved in metabolism were present in both the leaf and root (Fig. 3). More Cd-responsive genes were identified in the leaf than in the root, suggesting that different mechanisms exist at the molecular level in the leaf and root for dealing with conditions of Cd stress. Among the Cd-responsive genes, numerous genes involved in biotic stress signalling were activated, there included heat shock proteins (HSPs), glutathione S-transferase gene (GST), and glutathione (GSH) in the leaf (Additional file 2: Table S8, Fig. 3a), while most of the HSPs and all GST genes were induced in the root (Additional file 2: Table S9, Fig. 3b). A number of genes related to secondary metabolism and many genes implicated in signalling pathways were also significantly up-regulated in the leaf and root. Additionally, many of the cell wall related genes involved in biotic stress (Fig. 3a and b) and cell wall metabolism were highly expressed (Fig. 3c and d) in both the leaf and root.

### Differential expression of genes involved in cell wall metabolism and lignin synthesis in the leaf and root

**Fig. 4:**
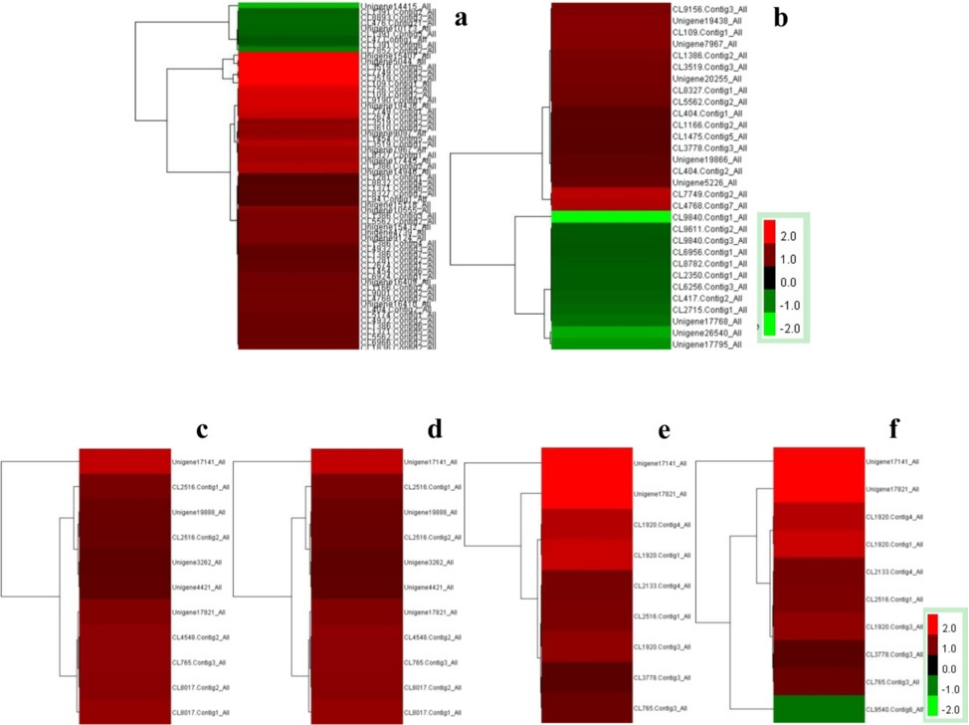
Genes involved in early response to Cd ions and expression profile of defence response by callose deposition in the cell wall, and cell wall thickening. **a** Cd-responsive genes in leaf, (**b**) Cd-responsive genes in root (**c**) Genes of callose deposition in the cell wall of the leaf. **d** Genes of cell wall thickening in the leaf. **e** Genes of callose deposition in the cell wall of the root. **f** Genes of cell wall thickening in the root

Many of the differentially expressed genes were up-regulated in both the leaf (Fig. 4a, Additional file 2: Table S10) and root (Fig. 4b, Additional file 2: Table S10) when exposed to Cd. Plants first absorb heavy metals through their root cell wall. Therefore, reducing influx across the cell wall is a key regulation switch. The expression profile of cell wall metabolism related genes were investigated. GO enrichment analysis revealed the expression of all genes involved in the defence response by callose deposition in the cell wall (Fig. 4c, Additional file 2: Table S10) and genes involved in cell wall thickening (Fig. 4d, Additional file 2: Table S10) were up-regulated in the leaf. Similarly, all genes involved in the defence response by callose deposition in the cell wall (Fig. 4e, Additional file 2: Table S10) and almost all differentially expressed genes involved in cell wall thickening (Fig. 4f, Additional file 2: Table S10) were up-regulated in the root.

**Fig. 5:**
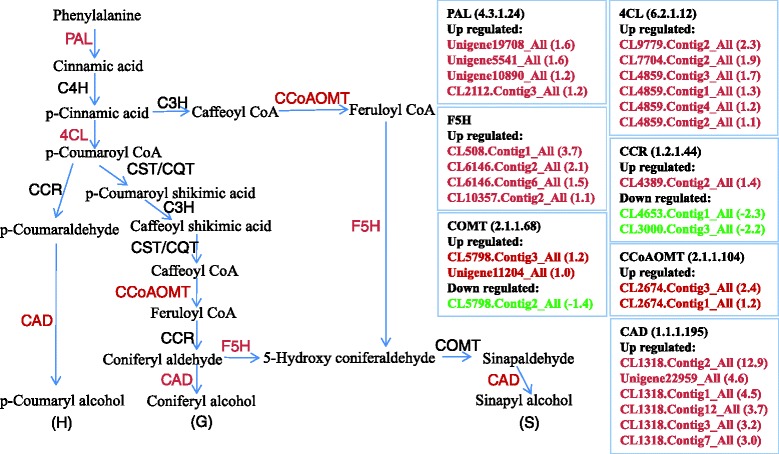
Biosynthesis pathway of lignin in the *S. matsudana* leaf after exposure to 50 μm Cd for 10 h

Genes coding for phenylalanine ammonia-lyase (PAL), 4-coumarate:coenzyme A ligase (4CL), ferulate-5-Hydroxylase (F5H), Coffee acyl coenzyme A-O-methyl transferase (CCoAOMT), and Cinnamyl alcohol dehydrogenase (CAD) showed elevated expression in the leaf (Fig. 5), but were not specifically expressed in the root. It is proposed that lignin content is specifically increased in the leaf, but not the root.

### Up-regulation of genes involved in sulphate assimilation, flavonoid metabolism and BRs biosynthesis in the leaf

**Fig. 6:**
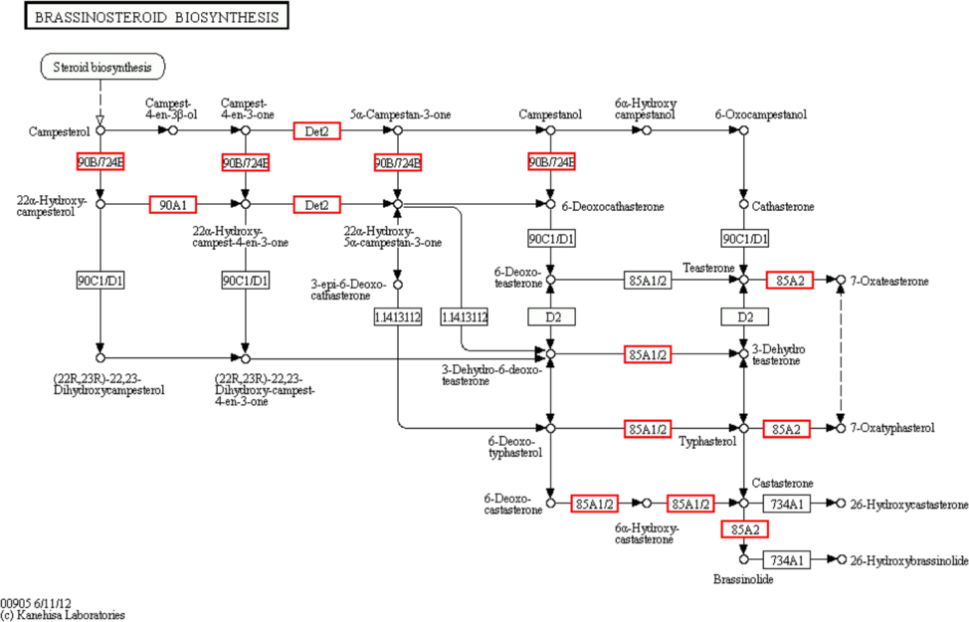
Metabolism pathways of brassinosteroid biosynthesis after exposure to 50 μm Cd for 10 h

As previously reported, sulphate assimilation and flavonoid metabolism respond to Cu or Cd [31]. In this study, numerous differentially expressed genes were involved in sulphate assimilation (Additional file 1: Figure S4) and metabolism of flavonoids (Additional file 1: Figure S5) in the leaf, almost all of these genes were up-regulated. In particular, numerous genes involved in BRs biosynthesis were mainly differentially expressed in the leaf, all of these were up-regulated (Fig. 6).

### Up-regulation of genes involved in metal transport and chelation binding in the root

ZIP transporters were highly expressed in roots in response to Cd (Additional file 2: Table S9, Fig. 3). Additionally, both sulphate and ABC transporters showed inducible higher expression levels in the leaf (Additional file 2: Table S8, Fig. 3) and root (Additional file 2: Table S9, Fig. 3). However, the expression levels of some known heavy metal resistant genes, including HMA, PC, and MT remained unchanged.

### Down –regulation of genes following cadmium exposure

Many genes were down-regulated upon Cd exposure, including members of the zinc finger family, cellulose synthase genes, and genes encoding arabinogalactan protein, xyloglucan endotransglycosylase and the auxin-response gene, indole-3-acetic acid (IAA) in the leaf (Additional file 2: Table S11), but only arabinogalactan protein and xyloglucan endotransglycosylase were inhibited in the root (Additional file 2: Table S12). While the gene synthesize IAA, was up-regulated in root (Additional file 2: Table S12).

### Validation of transcriptomic data by qRT-PCR

**Fig. 7:**
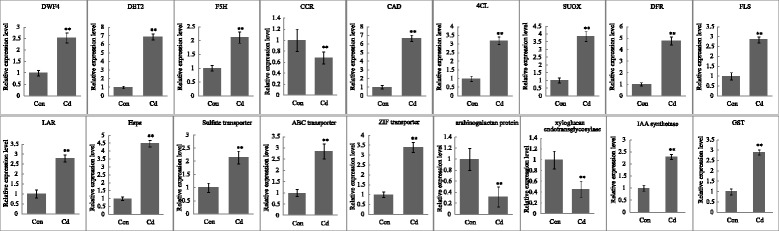
QRT-PCR analysis of selected genes. The means and standard error values from three independent samples are shown. Asterisks indicate significant differences between non- and Cd-treated leaves (ANOVA; *p* <0.01)

To validate the RNA-Seq data, 18 of the identified Cd tolerance related genes were verified by qRT-PCR. A total of 15 genes, including those encoding steroid 22-alpha-hydroxylase (DWF4), steroid 5-alpha-reductase (DET2), F5H, CAD, 4CL, sulfite oxidase (SUOX), flavanone 4-reductase (DER), flavonol synthase (FLS), leucoanthocyanidin reductase (LAR), Hsps, sulphate transporter, ABC transporter, ZIF transporter, IAA and GST) were up-regulated, while three genes, encoding CCR, arabinogalactan protein, and xyloglucan endotransglycosylase were down-regulated (Fig. 7). The qRT-PCR based expression profiles of the selected genes were similar to that detected using Illumina-sequencing based method, and thus supports our results.

### The cadmium transcriptome is linked with physiological changes

**Fig. 8:**
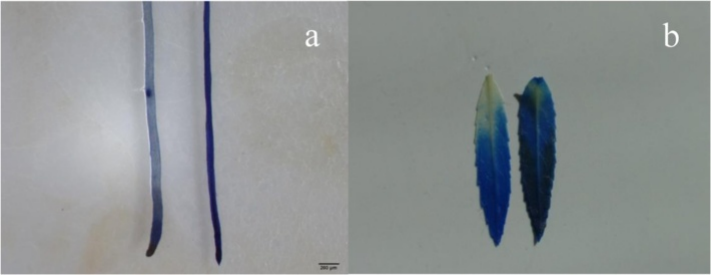
Callose staining. **a** Callose in the root of a control (*left*) and plant cultured in 1/2 MS medium supplemented with 50 μM Cd for 2 weeks (*right*). **b** Callose in the leaf of a control (*left*) and plant cultured in 1/2 MS medium supplemented with 50 μM Cd for 2 weeks (*right*)

Treatment of *S. matsudana* plants with 50 μM Cd for 2 weeks resulted in the induction of synthesis and deposition of callose in roots and leaves (Fig. 8). The blue colouring of the root (Fig. 8a, left) and leaf (Fig. 8b, left) in the control was lighter than that observed in the root (Fig. 8a, right) and leaf (Fig. 8b, right) of Cd treated plant.

**Fig. 9:**
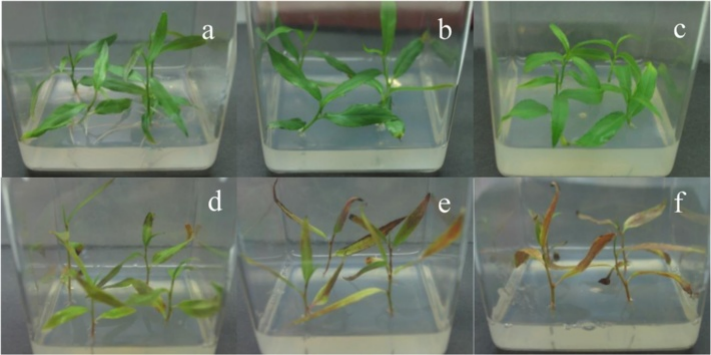
Effect of brassinazole (Brz) on *S. matsudana* in response to Cd. **a** Non-treated plant. **b** Seedlings cultured in 1/2 MS medium with the addition of 50 μM Cd for 2 weeks. **c** Seedlings cultured in 1/2 MS medium with the addition of 50 nM Brz for 2 weeks. **d** Seedlings cultured in 1/2 MS medium with the addition of 50 μM Cd + 10 nM Brz for 2 weeks. **e** Seedlings cultured in 1/2 MS medium with the addition of 50 μM Cd + 20 nM Brz for 2 weeks. **f** Seedlings cultured in 1/2 MS medium with the addition of 50 μM Cd + 50 nM Brz for 2 weeks

Plants grown under 50 μM Cd conditions showed similarly to the control plants (Fig. 9a and b) with green leaves observed in both control and Cd treated plants. Furthermore, the addition of Brz (BR inhibitor) had no significant affect upon plant growth (Fig. 9c). However, when grown under 50 μM Cd conditions, application of different concentrations of Brz affected growth (Fig. 9d–f). As concentration increased the plants became more sensitive to Cd, with leaves becoming yellow and falling off (Fig. 9f). This assay indicated that BRs directly play a central role in the early response to Cd in leaves.

**Fig. 10:**
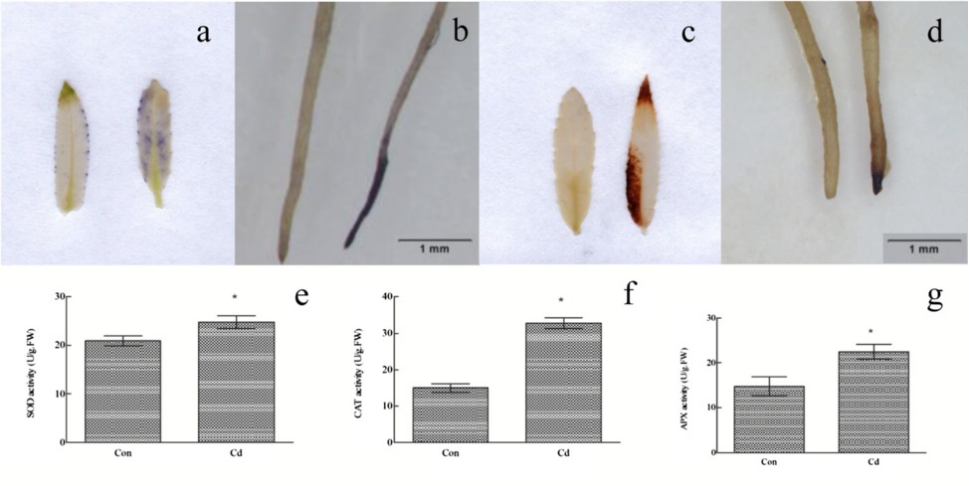
Measurement of reactive oxidative species levels and anti-oxidative enzymes activity. Detection of O_2_·^−^ in (**a**) leaf and (**b**) root of a control (*left*) and plant treated with 50 μM Cd for 10 h (*right*). Detection of H_2_O_2_ in (**c**) leaf and (**d**) root of a control (*left*) and plant treated with 50 μM Cd for 10 h (*right*). **e** Superoxide dismutase activity. **f** Catalase activity. **g** Ascorbate peroxidase activity. Data are mean ± SD from three independent experiments with three replicates. *Significant (*t* test, *P* <0.05) difference compared with WT plants under each treatment

In plants, Cd stress markedly induces ROS accumulation and affects antioxidant content [29]. Therefore, the generation of ROS was assayed. Leaves and roots were stained with NBT (a histochemical reagent for superoxide anions; Fig. 10a and b) and DAB (a histochemical reagent for H_2_O_2_; Fig. 10c and d). However, the ROS level was higher in the leaves and roots of the plants treated with Cd than that in control plants (Fig. 10a–d). Production of ROS was mainly observed in the root tip and the elongation zone (Fig. 10b and d). SOD, CAT, and APX activities were enhanced in the Cd-treated plant (Fig. 10e–g), indicating that antioxidant activity was activated in the early response to Cd.

## Discussion

### Vacuolar Sequestration in root cell and transcriptionic regeulation in leaf are main mechanisms in early response to Cd

This study investigated early effects of stress in the leaf and root of *S. matsudana* at high Cd concentration. Overall, the number of differentially expressed genes in the leaf was greater than in the root, and especially for up-regulated genes (Fig. 3). A large number of differentially regulated genes involving biological regulation and metabolism responses to Cd were different in the leaf (Fig. 3a and c) and root (Fig. 3b and d), probably owing to the lower Cd accumulation in the leaf (Fig. 2a). It was observed that some regulated genes or pathways were expressed in tissue-specific manner during the early response to Cd (10 h), leading to the development of different Cd defence mechanisms in the leaf and root.

Concentration analysis revealed that roots accumulated more Cd than other plant tissue (Fig. 2a). The results suggested that Cd only existed in the roots at an early stage, as Cd was transported to aboveground parts after 1 month of treatment. Metals are transported from roots to aboveground tissues via the xylem, and localize in aerial tissues; they are often compartmentalized or sequestrated in inactive photosynthetic tissues [32]. Cadmium compartmentalization in the vacuoles is common for herbaceous plants such as *Arabidopsis halleri* and *Thlaspi caerulescens* [32], and proven in *Populus* [29]. In the current study, sequestration in the vacuolar was also a strategy for *S. matsudana* to minimize cadmium damage.

Currently, no Cd-specific influx transporter has been reported for plant cells, however, transport can be realized by ZIP transporters, albeit at a low affinity; ZIP transporters have a highly specific transport affinity for Zn or Fe [33]. In the current study, ZIP transporters were highly expressed in the root (Additional file 2: Table S9, Fig. 3), suggesting that Cd was co-transported into the protoplast. PCs and glutathione (GSH)-derived metal-binding peptides are produced upon sulphate assimilation and are involved in metal detoxification; they are recognized as substrates and transported into the vacuole [9]. Generally, Cd is transported into the vacuolar in the form of a PC-Cd complex by ATP-binding cassette (ABC) transporters, and not as an independent Cd ion [34, 35]. GSH and ABC transporter genes were predominantly expressed (Tables S8 and S9, Fig. 3). In fact, the TEM result confirmed that Cd was segregated into the vacuole (Fig. 1e). We therefore speculate that it realize vacuole sequestration in *S. matsudana* by other pathways.

### Cell wall metabolism and lignin synthesis play a central role in early response to Cd

The cell wall is the first plant structure to come in contact with metals, and binding metal to the plant cell wall is a method of preventing metal entry into plants [36]. We observed root cell wall thickening following plant exposure to 50 μM Cd for 1 month (Fig. 1d). Furthermore, in the treated plants, granular deposits were observed in the intercellular space in the cortical part of the root (Fig. 1f); this phenomenon did not occur in the control plants (Fig. 1a and c).

Cell wall component polysaccharides can bind divalent and trivalent metal ions [37]. Callose (a polysaccharide) deposition in adjacent cell walls may effectively block symplastic transport of metal ions through the plasmodesmata [38]. This may protect the plant from a wide spread of toxic metal ions. Although the precise role of the induction of callose synthesis in plants exposed to toxic metal ions remains unclear, it is reported that the callose produced likely functions as a physical barrier to inhibit transport of metal ions from the apoplast to the symplast [39]. Increased callose synthesis has been reported in several plants when exposed to heavy metal, including *Lemna minor* L. [40] and poplar [41]. The induction of genes involved in callose deposition in the cell walls of leaves (Fig. 4c) and roots (Fig. 5e) illustrates that synthesis of callose deposition is a common strategy against stress factor penetration in plants. Indeed, callose was synthesized in both root and leaf following a longer period (2 weeks) of Cd treatment (Fig. 8). Likewise, differentially expressed genes involved in cell wall thickening were almost all up-regulated in the leaf (Fig. 4d) and root (Fig. 4f); this is consistent with results shown in Fig. 1d. It is more than possible that the cell wall thickening was a result of callose synthesis. Thus, thickening of the cell walls of the cortex is an alternative method to reduce uptake of unwanted metals during the early response to Cd in *S. matsudana*.

Some zinc finger proteins are demonstrated to be involved in abiotic and biotic stresses. Their zinc finger domains are proposed as mediators for toxic metals. Although these proteins are zinc-dependent, divalent metal residues, such as Cd, lead and copper are able to displace the zinc ion from the domain coordination core [42]. The replacement of zinc by extraneous Cd may contribute to the down-regulation of zinc finger family genes in the leaf. A previous study has proposed this replacement of zinc as a potential mechanism for uptake of the toxic metal. Cellulose is an essential component of both primary and secondary cell walls of higher plants, it is composed of a polymer of *β* (1, 4)-linked glucose. Xyloglucan is a major component in hemicellulose of plant cell walls. All cellulose synthase genes and xyloglucan endotransglycosylase genes were down-regulated in the leaf (Additional file 2: Table S11). Therefore, the cellulose and hemicellulose content of plant cell decreased under Cd stress. These results coincide with the fact that most genes involved in lignin synthesis were induced in response to Cd stress (Fig. 6). Arabinogalactan proteins are widely distributed in plant tissues and cells. They have been shown to play a role in the growth and development of higher plants, and especially in root regeneration [43]. The down-regulation of arabinogalactan proteins in the leaf and root (Additional file 2: Table S11) indicates that Cd also affected plant growth, though no morphological differences were observed that could further affect the expression level of arabinogalactan proteins. Additionally, all genes synthesized IAA were down-regulated in the leaf under Cd stress. IAA is a naturally abundant auxin with a regulatory function in plant growth [44]. In this study, IAA synthesis in the root but was inhibited when exposed to Cd. Application of IAA has been proven to increase shoot uptake and phytoextraction efficiency of Pb both in solution and soil [45]. It is proposed that up regulation of genes synthesize IAA in the root contributed to Cd phytoextraction from the root but not uptake to the leaf at the early stage of Cd stress.

Lignin content of plants can be affected by toxic heavy metals [46]. Occurrence of lignin enhancement may play a role in the physical barrier preventing Cd from entering the plant. Almost all key differentially expressed genes (e.g., *PAL, 4CL, CCoAOMT*, and *CAD*) in the lignin synthesis pathway were up-regulated in the leaf (Fig. 5), suggesting that Cd treatment induced increased lignin content. A similar result was found in response to Cd stress in the roots of other plants. When exposed to excess Cd conditions, lignin biosynthesis genes were up-regulated in the roots of *Noccaea caerulescens* and *A. thaliana* [47]. Cd could also induce synthesis of lignin in the roots of *Phragmites australis* [48]. Therefore, increased lignin content may contribute to Cd tolerance in the *S. matsudana* leaf.

### Sulphate assimilation and flavonoids metabolisms response to Cd

Basic metal tolerance mechanisms are similar in various plants. Lin *et al.* [31] reported that sulphate assimilation, flavonoids, GST, and HSPs play important roles in plant response to heavy metal stress. Similarly, in this study, GST, GSH, and HSP genes were all up-regulated in the leaf (Additional file 2: Table S8), and most genes involved in sulphate assimilation and flavonoid metabolism were also induced, thus indicating a complex Cd-tolerance network for early stress in the leaf. Genes involved in sulphur assimilation are always regulated upon exposure to heavy metals; this is because sulphur metabolism is a key pathway for the synthesis of molecules in response to heavy metals [49]. As it has a high metal binding affinity, GSH is able to work as a chelator [50]. Induction of HSPs in response to heavy metal stress is thought to prevent misfolding of proteins, protein aggregation, and the degradation of proteins under stress [51]. Cadmium stress can induce the expression of several *Arabidopsis* HSPs, including HSP70s and HSP60s [52].

### Brassinosteroids and antioxidative enzymes protect S. matsudana againt toCd at early stage

Brassinosteroids are a group of steroidal plant hormones accociated with a number of protective biotic and abiotic stress properties in plants. Previous studies revealed that BRs have the ability to lower metal uptake in plants, probably because of binding of BRs with membrane proteins that enhance metabolic activities by detoxifying heavy metal stresses [53]. In this study, all differentially expressed BR biosynthesis pathway genes in the leaf were up-regulated (Fig. 6). Furthermore, *S. matsudana* was more sensitive to Cd when BR was inhibited by the addition of Brz (Fig. 9d–f), suggesting BR involvement in early response to Cd in the *S. matsudana* leaf.

A sudden increase in reactive oxygen species leads to extremely toxic products, this invokes an early response to heavy metals, which may lead to lipid peroxidation, enzyme inactivation, and DNA and membrane damage [52]. However, this oxidative burst is required for downstream callose deposition in plants [53]. Cadmium exposure can provoke many different genes involved in scavenging ROS in plants [54]. Several studies have indicated that a high antioxidative capacity is responsible for heavy metal accumulation in plants [55]. The activities of SOD, CAT, and APX were highly activated in both the leaf and root under Cd stress (Fig. 10), suggesting that antioxidative enzymes play a role in the early response of *S. matsudana* to Cd.

## Conclusions

*S. matsudana* mainly accumulated Cd in the vacuole and intercellular space of the root cortex cell. It is noteworthy that the cell wall thickening occurred in the root cells. The transcriptional data was consistent with the result that genes involved in cell wall thickening and defence response by callose deposition in the cell wall were up-regulated in the root. This research provides a new insight into the understanding of Cd tolerance in woody tree species. Root-to-shoot transportation of Cd did not occur in the early response to Cd, suggesting that different mechanisms are involved in roots and leaves. Expression profile data revealed that transcriptional expression patterns were distinct in the leaf and root. A complex transcriptional network in the leaf triggers signal transduction, leading to tolerance to cadmium stress. All differentially expressed genes belonging to the BRs biosynthesis pathway were up-regulated in the leaf, suggesting that BRs are involved in the early response to Cd in the leaf. Additionally, the activation of antioxidative enzyme (SOD, CAT, and APX) activity, both in the root and leaf, suggests the presence of a physiological regulation mechanism to Cd exposure. We systematically investigated tolerance mechanisms in the root and leaf at substructure, transcriptional, and physiological levels. Our study provides useful information for understanding the molecular mechanisms involved in metal absorption, distribution, and transportation, and makes a significant contribution to determining tissue-specific responses to Cd stress.

## Availability of supporting data

The data sets supporting the results of this article are included within the article and additional files.

## Additional files

Additional file 1: Figure S1. Plant screening and growth. (a) Seeds cultured in 100 μM Cd medium for 1 month. (b) Plant growth when exposed to 50 μM Cd for 1 month. **Figure S2.** Measurement of chlorophyll and cadmium. (a) Chlorophyll a, b, and a + b content in the root, stem, and leaf of *S. matsudana* Koidz. (b) Cd concentration in the root, stem, and leaf treated with 50 μM Cd for 1 month. **Figure S3.** Characteristics of the similarity search of unigenes against Nr databases. (a) *E*-value distribution of BlastX hits for each unigene with an *E*-value threshold of 10*E* ^−5^. (b) Similarity distribution of the top BLAST hits for each unigene. (c) Species distribution is shown as a percentage of total homologous sequences with an *E*-value of at least 1.0*E* ^−5^. (d) Clusters of orthologous group functional classification of all unigenes. (e) GO classifications of assembled unigenes. **Figure S4.** Metabolism pathways of sulfur metabolism. **Figure S5.** Metabolism pathways of flavonoid biosynthesis. (PPT 732 kb) Additional file 2: Table S1. Unigene primers used for QRT-PCR analysis. **Table S2.** Sequencing output statistics. **Table S3.** Statistics of assembly quality. **Table S4.** Unigene classification by clusters of orthologous groups function. **Table S5.** Unigenes assembled by Gene Ontology classification. **Table S6.** Gene analysis by MapMan in the leaf. **Table S7.** Gene analysis by MapMan in the root. **Table S8.** Up-regulated genes in response to cadmium in leaf. **Table S9.** Up-regulated genes in response to cadmium in root. **Table S10.** Genes used for cluster analysis. **Table S11.** Down-regulated genes in response to cadmium in leaf. **Table S12.** Down-regulated genes in response to cadmium in root. (DOC 751 kb)

## Abbreviations

APX: Ascorbate peroxidase
BR: Brassinosteroid
Brz: Brassinazole
CAD: Cinnamyl alcohol dehydrogenase
CAT: Catalase
CCoAOMT: Coffee acyl coenzyme A-O-methyl transferase
Cd: Cadmium
4CL: 4-coumarate:coenzyme A ligase
CTAB: Hexadecyltrimethylammonium bromide
DER: Flavanone 4-reductase
DET2: Steroid 5-alpha-reductase
DGE: Digital gene expression
DWF4: Steroid 22-alpha-hydroxylase
F5H: Ferulate-5-Hydroxylase
FLS: Flavonol synthase
GO: Gene ontology
GSH: Glutathione
GST: Glutathione S-transferase gene
HMA: ATPase
HSPs: Heat shock proteins
IAA: Indole-3-acetic acid
LAR: Leucoanthocyanidin reductase
MTs: Metallothioneins
NGS: Next generation sequencing
PAL: Phenylalanine ammonia-lyase
PCs: Phytochelatins
PGRs: Plant growth regulators
RIN: RNA integration number
SOD: Superoxide dismutase
SUOX: Sulfite oxidase
