# Supplementary figures and images for: Characterization of early transcriptional responses to cadmium in the root and leaf of Cd-resistant Salix matsudana Koidz
Source: BMC Genomics. 2015 Sep 17;16(1):705. doi: 10.1186/s12864-015-1923-4 (PMC4573677; doi:10.1186/s12864-015-1923-4)

## Slide 1
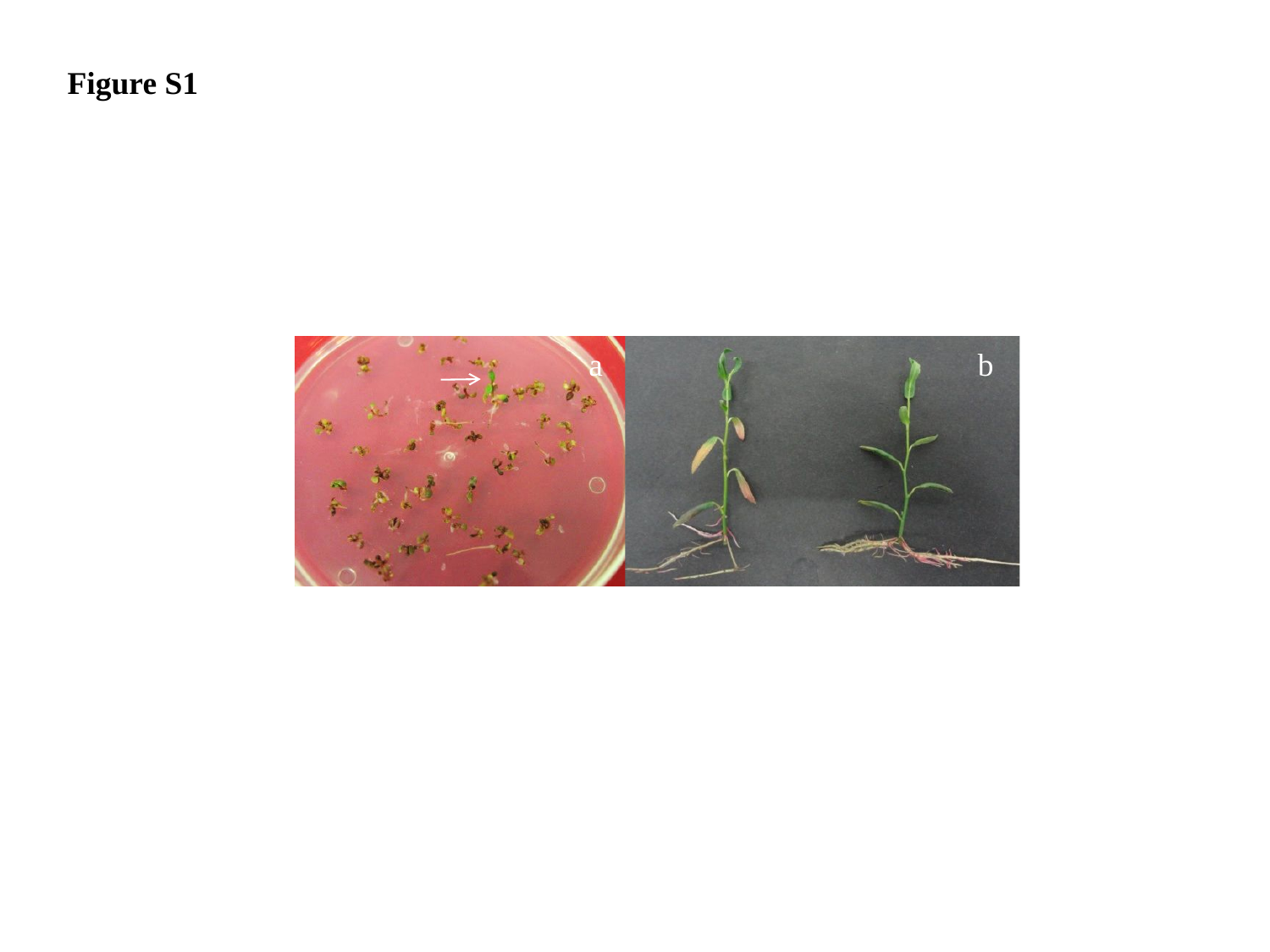

Figure S1
a
b

## Slide 2
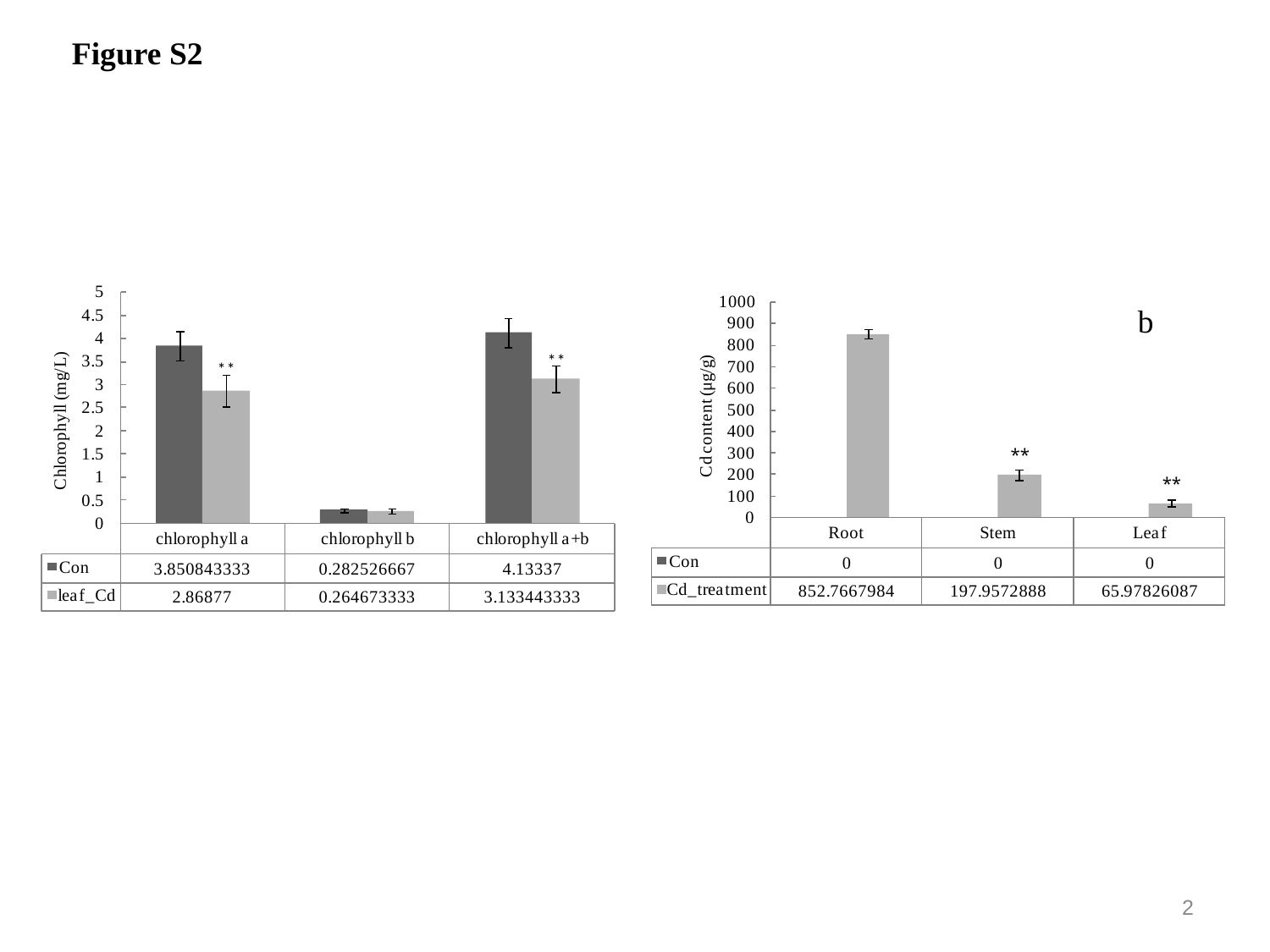

Figure S2
a
b
<number>

## Slide 3
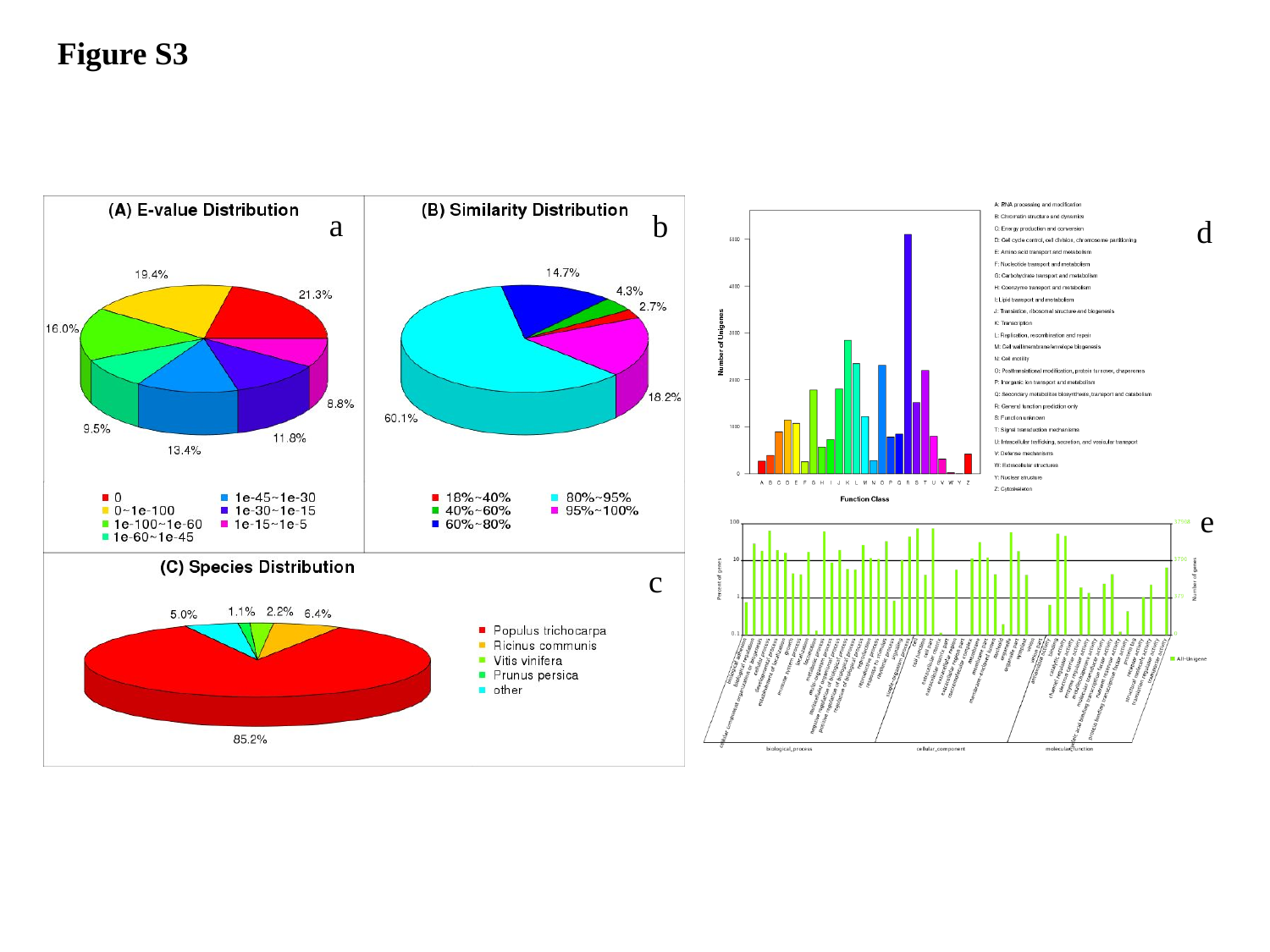

Figure S3
a
b
d
e
c

## Slide 4
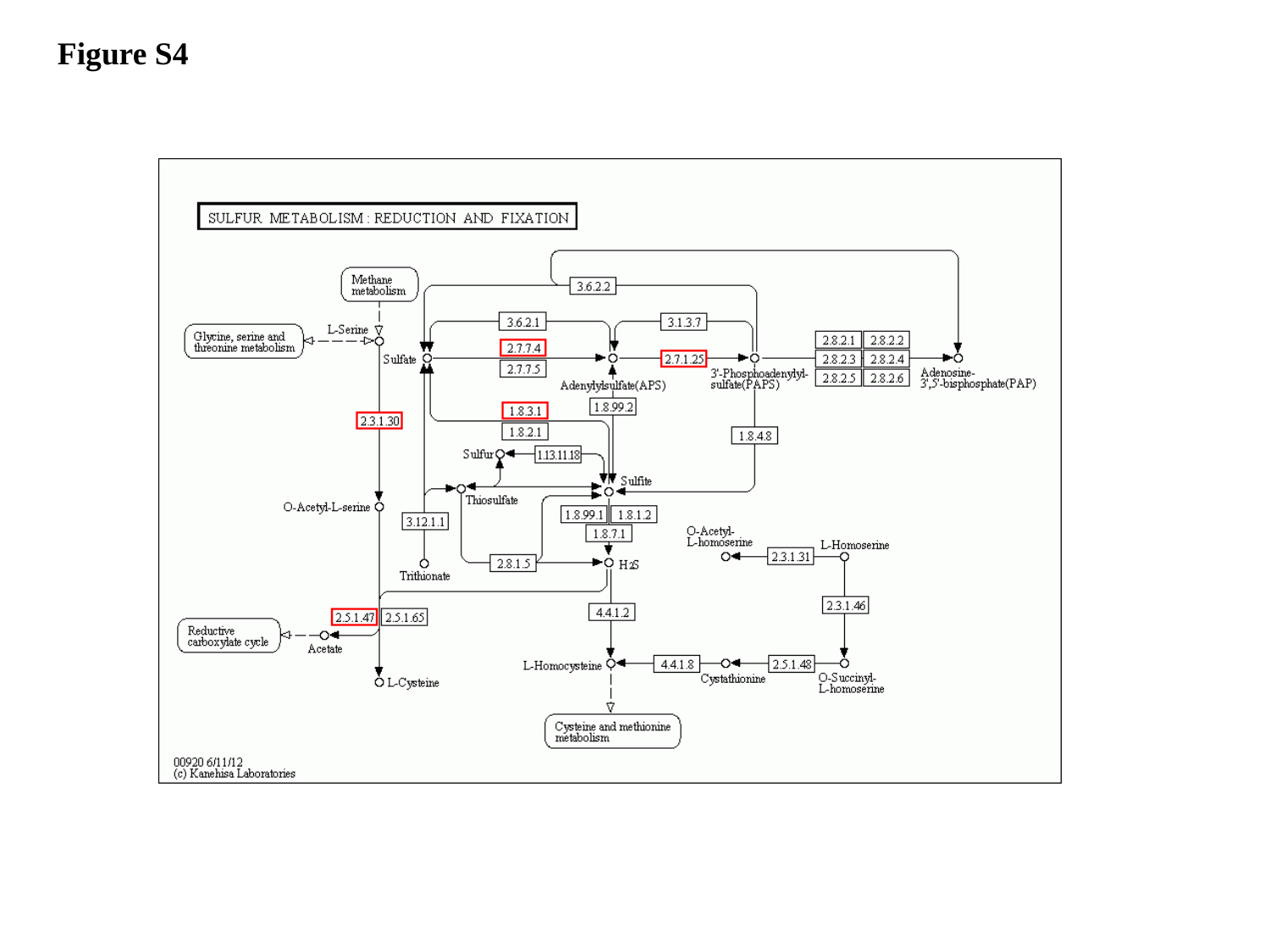

Figure S4

## Slide 5
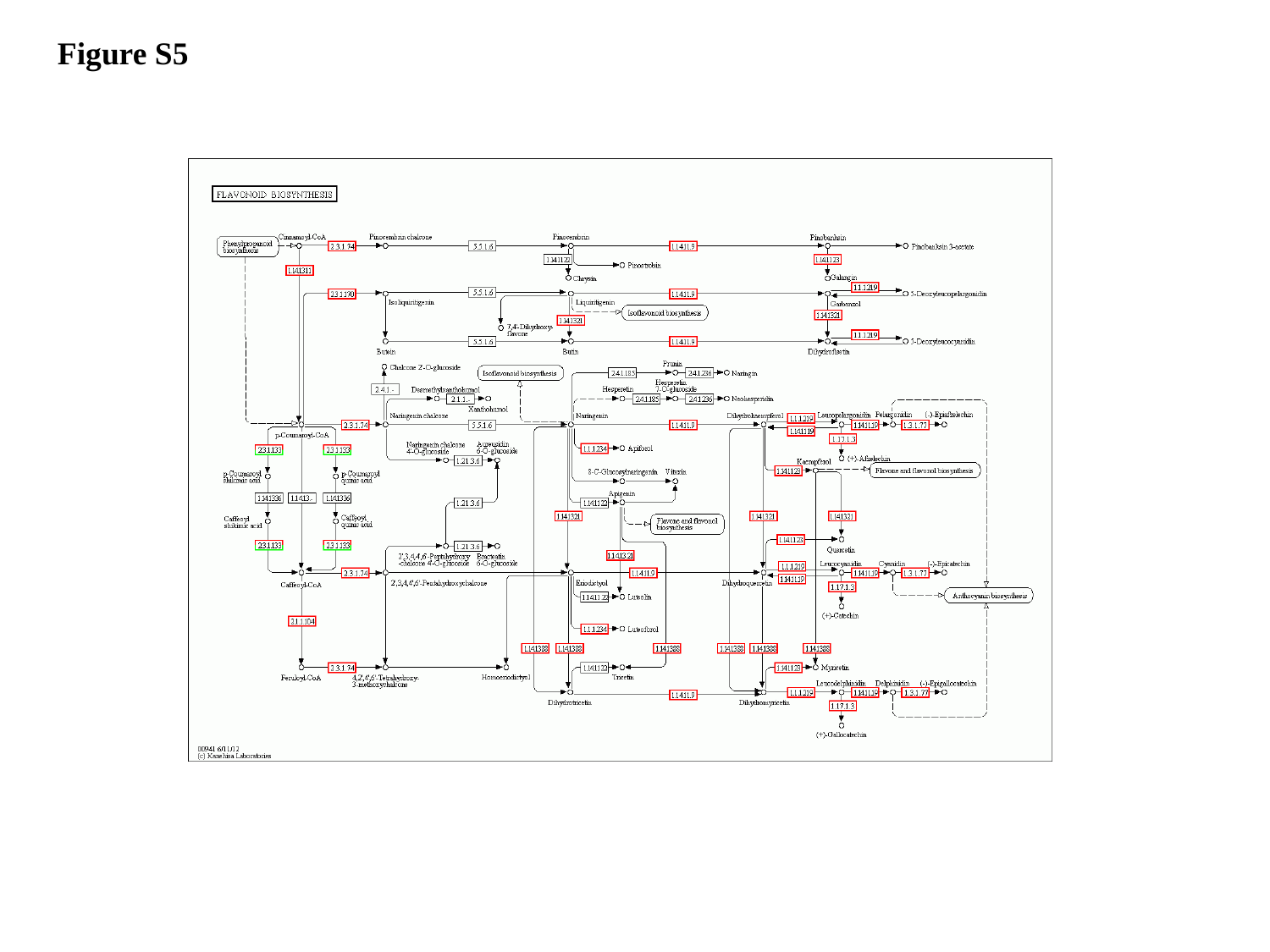

Figure S5

Supplement: Additional file 1: Figure S1. — Plant screening and growth. (a) Seeds cultured in 100 μM Cd medium for 1 month. (b) Plant growth when exposed to 50 μM Cd for 1 month. Figure S2. Measurement of chlorophyll and cadmium. (a) Chlorophyll a, b, and a + b content in the root, stem, and leaf of S. matsudana Koidz. (b) Cd concentration in the root, stem, and leaf treated with 50 μM Cd for 1 month. Figure S3. Characteristics of the similarity search of unigenes against Nr databases. (a) E-value distribution of BlastX hits for each unigene with an E-value threshold of 10E −5. (b) Similarity distribution of the top BLAST hits for each unigene. (c) Species distribution is shown as a percentage of total homologous sequences with an E-value of at least 1.0E −5. (d) Clusters of orthologous group functional classification of all unigenes. (e) GO classifications of assembled unigenes. Figure S4. Metabolism pathways of sulfur metabolism. Figure S5. Metabolism pathways of flavonoid biosynthesis. (PPT 732 kb) [file 12864_2015_1923_MOESM1_ESM.ppt]
