# Supplementary material for: Characterization of early transcriptional responses to cadmium in the root and leaf of Cd-resistant Salix matsudana Koidz
Source: BMC Genomics. 2015 Sep 17;16(1):705. doi: 10.1186/s12864-015-1923-4 (PMC4573677; doi:10.1186/s12864-015-1923-4)
Supplement: Additional file 2: Table S1. — Unigene primers used for QRT-PCR analysis. Table S2. Sequencing output statistics. Table S3. Statistics of assembly quality. Table S4. Unigene classification by clusters of orthologous groups function. Table S5. Unigenes assembled by Gene Ontology classification. Table S6. Gene analysis by MapMan in the leaf. Table S7. Gene analysis by MapMan in the root. Table S8. Up-regulated genes in response to cadmium in leaf. Table S9. Up-regulated genes in response to cadmium in root. Table S10. Genes used for cluster analysis. Table S11. Down-regulated genes in response to cadmium in leaf. Table S12. Down-regulated genes in response to cadmium in root. (DOC 751 kb) [file 12864_2015_1923_MOESM2_ESM.doc]

**Table S1.** Unigenes primers used for QRT-PCR analysis.

| **Unigenes name (Gene ID)** | **Primer sequence (5’-3’)** | **Product size (bp)** |
| --- | --- | --- |
| β-tubulin (CL476.Contig21_All) | F:CGATCTTCGAAAACTAGCCG  R: CATCCCACATTTGCTGTGTC | 143 |
| DWF4 (Unigene1172_All) | F: GCTTTCCTATCTGGTGCTGC  R: AATCAAGAACCACCCCACTG | 110 |
| DET2 (CL2797.Contig2_All) | F: CAAGCTGTTTGGAGCATTC  R: CCATATCGGTTTCAGCCACT | 156 |
| F5H (CL508.Contig1_All) | F: ACCGTAAATGGAGGGCTCTT  R: TGTGTGTCCACGATGAACCT | 185 |
| CCR (CL4653.Contig1_All) | F: CTTTCCTCGAGTCTTCAACA  R: GCAAGAAACAAGAAGCCGTC | 128 |
| CAD (CL1318.Contig2_All) | F: TGCTGATTCTCTCCGTTCCT  R: GTGTTGATGGCGTTGATGAC | 167 |
| 4CL (CL9779.Contig2_All) | F: CCGAGACCTTGTTCAGAAGC  R: CAAGCATGAGAAGACGGTG | 132 |
| SUOX (CL6329.Contig2_All) | F: TCTCAGGCTGGGTGTTAAGG  R: CACCCAGAGAAGCCCAAT | 139 |
| DFR (CL3116.Contig1_All) | F: ATGTTCGTAG  GTGCTGTGGA  R: GCAGGAGCCACTTCATTCTC | 105 |
| FLS  (CL8532.Contig1_All) | F: ACCCAACAGAACTCAAATTTC  R: GTTGGGACGAACGCAGTATT | 137 |
| LAR (CL7564.Contig2_All) | F:GCTGTTCCTGTAGAGCCAGG  R: GTGCCGTCACCGTAGATTTT | 191 |
| Hsps (CL8430.Contig1_All) | F: GCATTTGCCAACACGCGCAT  R: TCTTGATTTCCACTTTGGGC | 144 |
| Sulfate transporter (CL4689.Contig8_All) | F: TAGTCTTTGCATTCCCCAGG  R: CTTCTGCATTGCGTACAGG | 206 |
| ABC transporter (Unigene13614_All) | F:CGTCCCTTCAACATCATCAA  R:ACTTGTTTCCACAGCAAGGG | 187 |
| ZIF transporter (CL2446.Contig2_All) | F: TCGATGGAACGAGAATTTCC  R: CCTTCACCACCTGACCTTGT | 219 |
| AGPs (CL2728.Contig4_All) | F: CTTTAAATGGCCTCAGCCAG  R: CGTTGACTTGGTTGCTTTGA | 174 |
| XET (Unigene10610_All) | F: ACATGCAGCTGAAGTTGGTG  R: TGGGGTCGAACCAAAGATAG | 201 |
| IAA (Unigene1168_All) | F:CCAGGCATCTCCGTCATATT  R:AGTGCCAAGCATTGGAAGAT | 199 |
| GST (Unigene15407_All) | F:ATGCACCTCT  TGATCCATGC  R:TTGGATTTGTGGATGTGGTG | 121 |

**Table S2.** Sequencing output statistics.

| **Samples** | **Total raw reads** | **Total clean reads** | **Total clean nucleotides** | **Q20 percentage** | **N percentage** | **GC percentage** |
| --- | --- | --- | --- | --- | --- | --- |
| leaf | 28,571,826 | 26,468,532 | 2,382,167,880 | 97.51% | 0.00% | 44.87% |
| leaf_Cd | 30,368,658 | 27,760,428 | 2,498,438,520 | 97.59% | 0.00% | 44.91% |
| root | 29,621,130 | 26,476,324 | 2,382,869,160 | 97.16% | 0.00% | 48.73% |
| root_Cd | 28,621 ,130 | 25,873,264 | 2,328,593,760 | 96.99% | 0.00% | 48.87% |

**Table S3.** Statistics of assembly quality.

|  | **Sample** | **Total number** | **Total length (nt)** | **Mean length (nt)** | **N50** | **Total consensus sequences** | | **Distinct clusters** | **Distinct singletons** |
| --- | --- | --- | --- | --- | --- | --- | --- | --- | --- |
| Contig | leaf | 133,014 | 39,053,316 | 294 | 454 | - | - | | - |
|  | leaf_Cd | 134,797 | 39,749,353 | 295 | 456 | - | - | | - |
|  | root | 123,093 | 34,114,262 | 277 | 388 | - | - | | - |
|  | root_Cd | 121,037 | 32,372,720 | 267 | 370 | - | - | | - |
| Unigene | leaf | 59,735 | 35,726,729 | 598 | 996 | 59,735 | 25,409 | | 34,326 |
|  | leaf_Cd | 61,685 | 36,590,398 | 593 | 991 | 61,685 | 26,117 | | 35,568 |
|  | root | 55,587 | 28,940,962 | 521 | 771 | 55,587 | 21,957 | | 33,630 |
|  | root_Cd | 52,385 | 26,743,849 | 511 | 785 | 52,385 | 20,096 | | 32,289 |
|  | All | 56,483 | 49,167,526 | 870 | 1363 | 56,483 | 28,322 | | 28,161 |

**Table S4. Unigene classification by clusters of orthologous groups function.**

| **Code** | **Functional-categories** | **Gene-number** |
| --- | --- | --- |
| A | RNA processing and modification | 266 |
| B | Chromatin structure and dynamics | 388 |
| C | Energy production and conversion | 891 |
| D | Cell cycle control, cell division, chromosome partitioning | 1140 |
| E | Amino acid transport and metabolism | 1072 |
| F | Nucleotide transport and metabolism | 255 |
| G | Carbohydrate transport and metabolism | 1783 |
| H | Coenzyme transport and metabolism | 568 |
| I | Lipid transport and metabolism | 722 |
| J | Translation, ribosomal structure and biogenesis | 1810 |
| K | Transcription | 2847 |
| L | Replication, recombination and repair | 2349 |
| M | Cell wall/membrane/envelope biogenesis | 1218 |
| N | Cell motility | 279 |
| O | Posttranslational modification, protein turnover, chaperones | 2311 |
| P | Inorganic ion transport and metabolism | 782 |
| Q | Secondary metabolites biosynthesis, transport and catabolism | 842 |
| R | General function prediction only | 5101 |
| S | Function unknown | 1521 |
| T | Signal transduction mechanisms | 2202 |
| U | Intracellular trafficking, secretion, and vesicular transport | 796 |
| V | Defense mechanisms | 312 |
| W | Extracellular structures | 19 |
| Y | Nuclear structure | 5 |
| Z | Cytoskeleton | 415 |

**Table S5.** Unigenes assembled by Gene Ontology classification.

| **Ontology** | **Class** | **Number of all Unigene** |
| --- | --- | --- |
| Biology process | Biology adhesion | 287 |
|  | Biology regulation | 10853 |
|  | Cellular component organization or biogenesis | 6935 |
|  | Cellular process | 24080 |
|  | Developmental process | 7328 |
|  | Establishment of localization | 6120 |
|  | Growth | 1716 |
|  | Immune system process | 1589 |
|  | Localization | 6509 |
|  | Locomotion | 50 |
|  | Metabolic process | 23418 |
|  | Multi-organism process | 3377 |
|  | Multicellular organismal process | 7372 |
|  | Negative regulation of biological process | 2272 |
|  | Positive regulation of biological process | 2173 |
|  | Regulation of biological process | 10119 |
|  | Reproduction | 4485 |
|  | Reproductive process | 4239 |
|  | Response to stimulus | 12613 |
|  | Rhythmic process | 317 |
|  | Signaling | 3949 |
|  | Single-organism process | 16816 |
| Cellular component | Cell | 28052 |
|  | Cell junction | 1563 |
|  | Cell part | 28050 |
|  | Extracellular matrix | 44 |
|  | Extracellular matrix part | 11 |
|  | Extracellular region | 2165 |
|  | Extracellular region part | 37 |
|  | Macromolecular complex | 4302 |
|  | Membrane | 11924 |
|  | Membrane part | 4670 |
|  | Membrane-enclosed lumen | 1601 |
|  | Nucleoid | 73 |
|  | Organelle | 22405 |
|  | Organelle part | 6827 |
|  | Symplast | 1561 |
|  | Virion | 11 |
|  | Virion part | 11 |
| Molecular function | Antioxidant activity | 246 |
|  | Binding | 20407 |
|  | Catalytic activity | 17810 |
|  | Channel regulator activity | 3 |
|  | Electron carrier activity | 732 |
|  | Enzyme regulator activity | 514 |
|  | Metallochaperone activity | 8 |
|  | Molecular transducer activity | 899 |
|  | Nucleic acid binding transcription factor activity | 1642 |
|  | Nutrient reservoir activity | 47 |
|  | Protein binding transcription factor activity | 163 |
|  | Protein tag | 6 |
|  | Receptor activity | 397 |
|  | Structural molecule activity | 857 |
|  | Translation regulator activity | 3 |
|  | Transporter activity | 2460 |

**Table S6.** Gene analysis by MapMan in the leaf.

| **Gene ID (**[***Arabidopsis***](../../../../Administrator/AppData/Local/Yodao/DeskDict/frame/20140818133010/javascript:void(0)%3B) [***thaliana***](../../../../Administrator/AppData/Local/Yodao/DeskDict/frame/20140818133010/javascript:void(0)%3B)**)** | **Gene ID (*Salix matsudana*)** | **log2(leaf_Cd_FPKM/leaf_FPKM)** |
| --- | --- | --- |
| at4g16260 | CL10094.Contig1_All | 1.8433 |
| at5g48000 | CL10963.Contig2_All | 1.7199 |
| at5g02490 | CL1386.Contig3_All | 1.4433 |
| at4g08380 | CL1520.Contig1_All | 2.0322 |
| at4g08370 | CL1677.Contig3_All | 2.1241 |
| at1g76690 | CL3519.Contig3_All | 4.6435 |
| at1g75280 | CL8327.Contig1_All | 1.9292 |
| at4g21990 | CL9453.Contig1_All | 2.0045 |
| at3g57270 | CL9716.Contig1_All | 2.0519 |
| at5g67300 | Unigene14415_All | -2.2133 |
| at3g47540 | Unigene14498_All | 1.5406 |
| at2g43570 | Unigene14499_All | 1.4473 |
| at5g65040 | CL8017.Contig2_All | 1.6221 |
| at1g33030 | Unigene11204_All | 1.0233 |
| at5g02500 | CL1386.Contig4_All | 1.4091 |
| at4g34050 | CL2674.Contig3_All | 2.3699 |
| at5g20150 | CL9138.Contig1_All | -1.7358 |
| at4g30440 | CL4426.Contig1_All | -1.7835 |
| at4g18670 | CL9347.Contig1_All | -1.7809 |
| at2g16060 | Unigene5218_All | 1.3715 |
| at4g37410 | CL8017.Contig1_All | 1.754 |
| at5g42020 | CL6203.Contig4_All | 1.4915 |
| at3g13790 | CL315.Contig1_All | 1.2728 |
| at1g30760 | CL1907.Contig5_All | 1.8757 |
| at3g19680 | CL5981.Contig3_All | -4.4865 |
| at5g49460 | Unigene4581_All | 1.0866 |
| at5g35190 | CL9520.Contig2_All | 1.2655 |
| at4g34131 | CL80.Contig3_All | 3.1126 |
| at3g19010 | CL5903.Contig2_All | 1.3527 |
| at4g03210 | CL5123.Contig1_All | -1.467 |
| at1g50040 | CL5981.Contig2_All | -3.3598 |
| at4g14130 | CL53.Contig5_All | -4.2754 |
| at5g63790 | CL10967.Contig1_All | 1.6265 |
| at2g17230 | CL7277.Contig6_All | -2.4933 |
| at5g24313 | CL1677.Contig1_All | 1.8639 |
| at5g44680 | CL2823.Contig3_All | -2.8739 |
| at1g10020 | CL6292.Contig3_All | -2.0747 |
| at3g54810 | CL1232.Contig3_All | -1.5448 |
| at4g08950 | CL1447.Contig5_All | -2.0511 |
| at2g39710 | Unigene18894_All | -3.5658 |
| at5g14780 | CL1166.Contig2_All | 1.3087 |
| at5g54510 | CL1845.Contig2_All | -1.5488 |
| at5g16010 | CL2797.Contig2_All | 3.7833 |
| at5g59670 | CL10691.Contig2_All | -1.5701 |
| at5g54380 | CL2066.Contig1_All | -1.4031 |
| at5g22580 | Unigene3151_All | 1.7484 |
| at4g07960 | CL8346.Contig2_All | -1.6555 |
| at1g66150 | CL3623.Contig3_All | -1.0635 |
| at1g60810 | CL2362.Contig1_All | 2.2722 |
| at4g11820 | CL1257.Contig2_All | 1.7304 |
| at4g11930 | Unigene14364_All | 1.0275 |
| at2g41690 | CL5174.Contig1_All | 1.271 |
| at5g26220 | Unigene15432_All | 1.4399 |
| at5g54160 | CL5798.Contig3_All | 1.2463 |
| at5g48230 | CL9680.Contig2_All | 1.4633 |
| at3g46120 | Unigene4316_All | 1.4384 |
| at5g43940 | CL2166.Contig1_All | 1.8363 |
| at4g15550 | Unigene2057_All | 4.1135 |
| at1g10670 | CL2362.Contig2_All | 1.1689 |
| at2g29460 | Unigene10879_All | 1.0128 |
| at5g19640 | CL756.Contig2_All | 2.6287 |
| at3g23240 | Unigene24104_All | 4.5706 |
| at3g21800 | CL2575.Contig2_All | 1.1213 |
| at4g35100 | Unigene9262_All | -1.3134 |
| at5g64250 | Unigene17445_All | 1.8812 |
| at3g47420 | CL1076.Contig2_All | -1.5432 |
| at4g17030 | Unigene562_All | 2.9304 |
| at3g02360 | CL8281.Contig5_All | 1.4732 |
| at4g23400 | Unigene2338_All | -1.4834 |
| at2g22250 | CL2155.Contig1_All | 2.2601 |
| at5g51550 | CL3375.Contig2_All | -2.5664 |
| at3g22460 | CL4768.Contig7_All | 1.3465 |
| at3g18000 | CL7571.Contig1_All | 1.5752 |
| at5g48740 | CL9771.Contig2_All | 1.7373 |
| at3g43800 | CL8086.Contig1_All | 2.8182 |
| at4g15230 | CL4548.Contig2_All | 1.6519 |
| at1g14550 | CL7562.Contig1_All | 5.1937 |
| at3g23800 | CL404.Contig2_All | 1.3548 |
| at2g02000 | CL3610.Contig2_All | 1.7639 |
| at4g23430 | CL10791.Contig1_All | 1.1111 |
| at4g35630 | CL6924.Contig1_All | 1.3238 |
| at4g38210 | CL10192.Contig1_All | -2.5635 |
| at5g06640 | CL9520.Contig4_All | 2.1013 |
| at4g13340 | CL6125.Contig2_All | -1.8343 |
| at3g61210 | CL7778.Contig3_All | 1.6776 |
| at5g58390 | CL8265.Contig2_All | 2.0068 |
| at3g16180 | Unigene409_All | -1.085 |
| at2g44670 | CL3972.Contig1_All | -1.2101 |
| at5g47770 | Unigene19510_All | 1.1825 |
| at4g25200 | CL9190.Contig1_All | 2.542 |
| at1g62660 | Unigene19593_All | -1.0154 |
| at3g01420 | Unigene19566_All | 1.2637 |
| at5g67450 | CL9796.Contig1_All | 1.2512 |
| at5g13930 | Unigene9150_All | 1.3923 |
| at5g13750 | CL3267.Contig3_All | 1.6945 |
| at1g23030 | CL1592.Contig2_All | -1.5187 |
| at1g76530 | Unigene4520_All | 1.3982 |
| at2g04240 | Unigene13347_All | -1.114 |
| at3g54250 | CL656.Contig3_All | 1.2679 |
| at3g45970 | CL2588.Contig3_All | -1.3549 |
| at5g18890 | CL10091.Contig1_All | 2.9725 |
| at5g59190 | CL418.Contig1_All | -1.0973 |
| at4g37990 | CL1318.Contig1_All | 4.5457 |
| at5g19890 | CL4.Contig1_All | 1.8507 |
| at5g35643 | CL9054.Contig2_All | -2.4882 |
| at3g11700 | CL8736.Contig4_All | -1.8424 |
| at5g05250 | Unigene10876_All | 1.3584 |
| at5g12270 | Unigene2315_All | 1.7012 |
| at1g19025 | CL883.Contig1_All | -1.7549 |
| at3g62100 | CL1370.Contig3_All | -2.2738 |
| at5g03380 | CL3835.Contig3_All | 1.5907 |
| at1g04100 | CL10834.Contig2_All | -1.3348 |
| at1g80330 | Unigene12785_All | 1.3643 |
| at1g63840 | CL1567.Contig1_All | -2.6044 |
| at5g56320 | Unigene7110_All | -1.7051 |
| at5g20950 | CL1364.Contig3_All | 1.7893 |
| at4g14390 | CL7735.Contig3_All | -1.5311 |
| at1g27140 | CL10219.Contig2_All | 1.849 |
| at3g59820 | CL8779.Contig1_All | 1.2509 |
| at2g30490 | CL9862.Contig1_All | 1.403 |
| at2g23210 | CL750.Contig1_All | 1.666 |
| at5g41890 | Unigene14347_All | 1.2188 |
| at4g38830 | CL78.Contig4_All | 2.2097 |
| at1g51830 | Unigene10441_All | 1.3816 |
| at1g72100 | Unigene4713_All | -1.7912 |
| at3g17790 | CL8856.Contig1_All | -1.4703 |
| at3g62660 | CL8310.Contig1_All | -1.0732 |
| at4g01830 | CL2946.Contig5_All | 1.0915 |
| at1g75620 | Unigene4716_All | 2.4313 |
| at2g43200 | CL1540.Contig7_All | -1.1498 |
| at1g36810 | Unigene10607_All | 1.056 |
| at1g80820 | CL4389.Contig2_All | 1.3919 |
| at4g37260 | CL3936.Contig3_All | -1.4223 |
| at2g47440 | CL3046.Contig3_All | -2.1621 |
| at1g27130 | CL10219.Contig1_All | 2.8188 |
| at4g24290 | CL184.Contig2_All | 1.0323 |
| at5g40590 | Unigene2572_All | 1.2813 |
| at1g13250 | CL2029.Contig2_All | -2.7577 |
| at3g52370 | CL8736.Contig2_All | -1.8397 |
| at4g38860 | Unigene18246_All | -1.3075 |
| at1g29430 | Unigene8036_All | -2.0908 |
| at4g27670 | CL1135.Contig2_All | 4.1639 |
| at1g71695 | CL1919.Contig1_All | 1.1586 |
| at4g34200 | CL2579.Contig1_All | 1.9744 |
| at3g49220 | CL7075.Contig1_All | -1.128 |
| at2g26730 | CL4027.Contig4_All | -1.1887 |
| at4g04490 | CL6109.Contig2_All | 1.5945 |
| at5g63390 | Unigene4527_All | -1.0094 |
| atmg01040 | Unigene8971_All | -2.5651 |
| at2g15130 | CL4898.Contig1_All | 1.7729 |
| at3g46230 | Unigene12934_All | 3.3093 |
| at5g64660 | Unigene6664_All | 1.1198 |
| at5g22940 | Unigene279_All | -1.1412 |
| at4g16160 | Unigene18149_All | -2.4856 |
| at1g27210 | CL2197.Contig2_All | -1.1039 |
| at3g59010 | CL8564.Contig1_All | 1.4101 |
| at4g31550 | Unigene6614_All | 1.1914 |
| at5g48190 | CL1477.Contig2_All | -1.3034 |
| at2g25020 | CL7859.Contig1_All | -1.6258 |
| at2g29940 | Unigene8466_All | 2.7427 |
| at5g44360 | Unigene14649_All | 1.1483 |
| at3g11410 | CL1574.Contig1_All | -1.1567 |
| at4g33970 | CL4192.Contig2_All | -2.6674 |
| at4g16660 | CL3697.Contig2_All | 1.1568 |
| at4g21200 | CL97.Contig1_All | 2.1538 |
| at3g49820 | CL1949.Contig2_All | -1.45 |
| at5g13420 | CL1281.Contig1_All | 1.0017 |
| at1g52970 | CL8511.Contig1_All | -1.3911 |
| at3g28740 | Unigene17141_All | 2.2638 |
| at1g35140 | CL1447.Contig3_All | -2.0452 |
| at3g12710 | CL970.Contig2_All | -1.2811 |
| at3g23730 | CL53.Contig3_All | -2.8388 |
| at5g23380 | Unigene8267_All | -2.6682 |
| at2g15020 | CL7180.Contig1_All | 1.1763 |
| at1g30720 | CL3504.Contig2_All | 1.1656 |
| at1g63180 | CL10105.Contig1_All | -1.0514 |
| at1g34260 | CL1823.Contig4_All | -1.1559 |
| at4g35750 | CL910.Contig3_All | -1.4063 |
| at2g30770 | Unigene6914_All | 1.6219 |
| at4g16780 | CL3748.Contig2_All | -2.1972 |
| at4g19460 | Unigene12533_All | 1.9475 |
| at4g20900 | Unigene15089_All | 1.0427 |
| at2g14510 | Unigene12863_All | 1.8936 |
| at5g55360 | Unigene415_All | 1.8162 |
| at5g13760 | CL10622.Contig2_All | -1.2793 |
| at3g61440 | CL7293.Contig5_All | 1.1989 |
| at5g57530 | CL2071.Contig2_All | -2.2711 |
| at5g05600 | CL5751.Contig1_All | 1.9487 |
| at2g20800 | CL773.Contig3_All | 1.1712 |
| at2g20520 | CL2728.Contig2_All | -1.6051 |
| at3g59140 | CL5160.Contig2_All | 1.192 |
| at3g21780 | CL2575.Contig3_All | 1.2732 |
| at2g19200 | CL2180.Contig3_All | 1.4623 |
| at5g65800 | Unigene16131_All | 2.4529 |
| at4g02380 | CL4756.Contig1_All | 1.1356 |
| at3g15540 | CL2493.Contig2_All | -1.004 |
| at3g09790 | CL2269.Contig5_All | 1.6762 |
| at4g29720 | CL8768.Contig1_All | -1.54 |
| at3g44350 | Unigene15127_All | -1.3002 |
| at5g51990 | CL8942.Contig1_All | -2.4517 |
| at4g13090 | CL2071.Contig1_All | -2.3844 |
| at5g45340 | CL1539.Contig3_All | -1.8726 |
| at2g25090 | CL8460.Contig1_All | -1.4641 |
| at5g25910 | CL1168.Contig2_All | 1.116 |
| at2g38310 | CL6495.Contig1_All | 1.4644 |
| at5g05440 | CL6495.Contig2_All | 1.5006 |
| at3g63250 | CL6750.Contig2_Al | 1.2465 |
| at5g03860 | Unigene21326_All | -2.9241 |
| at5g41080 | CL6625.Contig3_All | -1.415 |
| at5g14060 | CL5856.Contig1_All | 1.0784 |
| at5g64990 | Unigene658_All | 1.7611 |
| at5g18060 | Unigene10023_All | -1.2231 |
| at1g28430 | CL1326.Contig4_All | 1.7012 |
| at3g06260 | CL5821.Contig1_All | -1.8992 |
| at3g22890 | CL5562.Contig3_All | 1.261 |
| at1g61100 | CL1387.Contig4_All | -1.2197 |
| at5g09800 | CL6253.Contig2_All | 1.1714 |
| at4g35010 | CL5880.Contig2_All | -1.1374 |
| at3g60390 | CL10482.Contig1_All | -1.7277 |
| at3g48850 | Unigene4659_All | 1.8493 |
| at1g08630 | CL2722.Contig2_All | 1.3833 |
| at4g13940 | CL94.Contig1_All | 1.0343 |
| at1g18140 | CL135.Contig1_All | 2.9908 |
| at5g14930 | CL4248.Contig3_All | 1.3064 |
| at5g51630 | CL1391.Contig2_All | -1.1625 |
| at5g66490 | Unigene3142_All | 2.1831 |
| at5g16110 | Unigene197_All | -1.4254 |
| at3g47170 | Unigene19090_All | -1.7513 |
| at2g02220 | CL3468.Contig4_All | 1.1343 |
| at3g21240 | CL4859.Contig3_All | 1.6746 |
| at1g06160 | CL8580.Contig1_All | 3.5706 |
| at2g12990 | CL4397.Contig2_All | -1.3886 |
| at1g07240 | CL7933.Contig4_All | 1.246 |
| at3g47110 | CL3749.Contig2_All | 2.2245 |
| at5g06800 | CL1800.Contig3_All | 2.017 |
| at5g24590 | CL807.Contig1_All | -1.1079 |
| at4g14560 | CL7275.Contig2_All | -1.5829 |
| at1g60060 | Unigene15873_All | -1.5672 |
| at4g39390 | CL2925.Contig3_All | 1.116 |
| at2g37770 | Unigene14946_All | 2.0086 |
| at1g71020 | CL1592.Contig3_All | -1.184 |
| at5g52900 | Unigene11233_All | -2.1409 |
| at1g11545 | CL2311.Contig2_All | -1.8587 |
| at4g08150 | CL8292.Contig2_All | -2.6581 |
| at5g25880 | Unigene13291_All | 1.0979 |
| at1g80160 | Unigene7100_All | 1.1855 |
| at5g17300 | CL2735.Contig2_All | -1.2218 |
| at2g35470 | Unigene10959_All | -1.0024 |
| at3g57920 | Unigene8652_All | -2.3059 |
| at4g01470 | CL3070.Contig2_All | 1.2678 |
| at3g43860 | CL3061.Contig3_All | 1.0691 |
| at3g20210 | CL10261.Contig2_All | -1.063 |
| at4g19230 | CL1539.Contig2_All | -1.5281 |
| at1g44830 | CL113.Contig2_All | -2.3171 |
| at3g51240 | CL5086.Contig1_All | 1.2455 |
| at5g57500 | Unigene15193_All | 3.3642 |
| at4g10490 | Unigene6579_All | 2.1538 |
| at2g23200 | CL1391.Contig6_All | -1.0787 |
| at2g23810 | CL1448.Contig1_All | -1.2107 |
| at5g44380 | Unigene6236_All | 1.3095 |
| at3g54420 | CL9594.Contig1_All | 1.439 |
| at4g32860 | CL9848.Contig1_All | -1.4977 |
| at4g24400 | CL1592.Contig1_All | -1.2276 |
| at5g36130 | CL10181.Contig1_All | 1.5028 |
| at4g00820 | CL1826.Contig1_All | -1.1449 |
| at5g09890 | CL1836.Contig2_All | 1.2893 |
| at4g38400 | CL2588.Contig1_All | -1.0023 |
| at5g20940 | CL1364.Contig1_All | 1.7173 |
| at5g66920 | Unigene18884_All | -1.2432 |
| at5g10980 | Unigene4379_All | 1.1981 |
| at5g17650 | Unigene19563_All | 1.1386 |
| at3g28580 | CL4145.Contig4_All | 2.1284 |
| at3g53780 | CL3206.Contig1_All | 1.8998 |
| at4g12860 | CL7531.Contig1_All | -1.2062 |
| at1g78380 | Unigene5044_All | 3.9566 |
| at5g61350 | CL8011.Contig2_All | -1.2412 |
| at1g62440 | Unigene9040_All | -2.5543 |
| at3g04690 | CL6102.Contig2_All | -1.0606 |
| at2g38060 | CL4843.Contig2_All | 2.2667 |
| at1g28130 | CL1845.Contig1_All | -2.5327 |
| at5g39700 | Unigene14749_All | 2.5759 |
| at1g61390 | CL9633.Contig2_All | 2.0065 |
| at4g11850 | CL4932.Contig3_All | 1.1633 |
| at2g29440 | CL2625.Contig2_All | 1.973 |
| at3g57070 | CL6451.Contig2_All | -1.1488 |
| at1g24140 | CL7258.Contig3_All | 1.015 |
| at1g67830 | CL2897.Contig3_All | 1.0903 |
| at5g28360 | CL10839.Contig1_All | 5.2122 |
| at5g07850 | Unigene6569_All | 1.4608 |
| at3g10960 | CL7317.Contig1_All | 1.2881 |
| at5g19380 | CL9001.Contig2_All | 1.3124 |
| at2g42380 | CL10192.Contig2_All | -2.1107 |
| at3g16150 | Unigene15161_All | 2.2122 |
| at2g01900 | Unigene6473_All | 1.2747 |
| at4g21230 | CL11033.Contig1_All | 1.5721 |
| at4g28670 | CL11080.Contig7_All | 1.0143 |
| at5g24090 | CL4368.Contig1_All | 3.4812 |
| at5g38260 | CL6854.Contig3_All | -1.2107 |
| at3g43600 | CL10236.Contig2_All | 1.0337 |
| at5g15240 | Unigene16968_All | 2.1248 |
| at1g30560 | CL1076.Contig1_All | -1.0961 |
| at4g39230 | CL8327.Contig2_All | 1.0347 |
| at2g15220 | CL4898.Contig3_All | 1.4186 |
| at5g05340 | Unigene19174_All | 2.4673 |
| at5g59220 | Unigene382_All | -1.2162 |
| at4g34650 | Unigene10363_All | 1.5 |
| at5g10930 | CL8460.Contig2_All | -1.2576 |
| at1g65840 | CL575.Contig1_All | -1.8157 |
| at1g28160 | Unigene10805_All | 1.6923 |
| at5g28490 | CL5383.Contig1_All | -1.7577 |
| at1g19250 | Unigene12087_All | 3.1896 |
| at2g27510 | CL957.Contig1_All | 1.9624 |
| at3g59080 | CL3577.Contig1_All | 1.4997 |
| at5g54860 | Unigene3326_All | 5.0562 |
| at2g23860 | CL4254.Contig3_All | -1.0177 |
| at2g45930 | CL4789.Contig1_All | -1.401 |
| at4g14550 | CL8893.Contig3_All | -1.2126 |
| at5g13380 | CL4401.Contig1_All | -1.0893 |
| at1g53680 | CL4652.Contig2_All | 1.443 |
| at2g03730 | CL903.Contig3_All | -1.1263 |
| at3g50340 | CL684.Contig2_All | -1.2331 |
| at4g25220 | CL1076.Contig3_All | -1.0425 |
| at3g53880 | Unigene15118_All | 1.0223 |
| at4g25720 | Unigene457_All | 1.0736 |
| at5g61890 | Unigene4741_All | 2.1284 |
| at4g34410 | CL4274.Contig5_All | 1.6346 |
| at5g58620 | CL10978.Contig1_All | 1.1702 |
| at5g48500 | Unigene5063_All | -1.6747 |
| at1g11280 | CL9123.Contig1_All | 1.1051 |
| at1g11170 | CL7190.Contig2_All | 1.2436 |
| at3g61460 | CL1567.Contig2_All | -2.1728 |
| at1g59740 | CL6129.Contig3_All | 1.0015 |
| at1g06980 | Unigene15169_All | -1.7737 |
| at1g72580 | CL8300.Contig2_All | 1.5561 |
| at3g23750 | CL7795.Contig1_All | -1.1786 |
| at3g55790 | Unigene16343_All | 4.8811 |
| at3g61580 | CL385.Contig3_All | -1.2576 |
| at1g51940 | CL9098.Contig2_All | -1.0825 |
| at5g25930 | CL9855.Contig1_All | 2.3598 |
| at5g62470 | CL47.Contig1_All | -1.0827 |
| at5g25430 | Unigene18972_All | 2.674 |
| at4g34138 | CL80.Contig2_All | 1.1181 |
| at4g08620 | CL4689.Contig6_All | 1.2115 |
| at5g54300 | CL10620.Contig1_All | 3.5117 |
| at2g37130 | Unigene12772_All | 2.0737 |
| at4g25480 | Unigene3292_All | -1.8697 |
| at5g04390 | CL7815.Contig1_All | -1.3 |
| at4g18170 | CL6043.Contig4_All | 1.1855 |
| at5g59120 | Unigene8622_All | 2.0502 |
| at5g12470 | CL5231.Contig1_All | 1.9054 |
| at2g18162 | CL9322.Contig2_All | 1.9492 |
| at5g49100 | Unigene8413_All | -1.3335 |
| at2g23620 | Unigene759_All | 1.1747 |
| at4g28530 | CL3459.Contig2_All | -2.9608 |
| at5g40960 | CL8145.Contig1_All | -1.1988 |
| at1g02900 | CL600.Contig1_All | 2.0263 |
| at3g09820 | CL8832.Contig4_All | 1.0067 |
| at2g39870 | CL1427.Contig4_All | -2.6234 |
| at5g40150 | CL6467.Contig2_All | -1.0352 |
| at4g31940 | Unigene16838_All | 1.1492 |
| at5g52390 | CL5996.Contig1_All | 2.0593 |
| at5g44110 | CL8462.Contig3_All | 1.2189 |
| at5g46690 | CL4856.Contig1_All | -1.1728 |
| at1g53903 | CL4844.Contig1_All | -1.2601 |
| at3g23820 | Unigene2576_All | -1.0166 |
| at5g17860 | Unigene15090_All | 1.3612 |
| at5g47650 | CL8614.Contig1_All | -1.025 |
| at1g17870 | Unigene10508_Al | -1.1745 |
| at5g60890 | Unigene13531_All | 3.8337 |
| at4g35320 | Unigene12714_All | -2.4655 |
| at2g21480 | CL8011.Contig1_All | -1.0525 |
| at4g02130 | Unigene8266_All | -1.272 |
| at4g32650 | Unigene18725_All | 1.4059 |
| at3g12700 | CL1163.Contig5_All | -1.5006 |
| at1g22340 | CL2307.Contig1_All | 1.5897 |
| at1g17750 | CL581.Contig3_All | -1.5278 |
| at5g48840 | Unigene19058_All | 1.4611 |
| at4g25760 | CL433.Contig2_All | -1.482 |
| at2g10940 | CL2497.Contig1_All | -1.2025 |
| at5g42180 | CL10448.Contig1_All | -2.6581 |
| at4g19120 | CL1540.Contig6_All | -1.085 |
| at5g23660 | Unigene19630_All | -4.5964 |
| at4g27030 | CL5272.Contig1_All | 1.4438 |
| at1g70900 | Unigene14756_All | -1.0109 |
| at1g74360 | Unigene6284_All | 1.0092 |
| at3g61260 | CL2013.Contig3_All | -1.1114 |
| at5g57560 | Unigene446_All | -1.4753 |
| at1g65790 | CL11080.Contig2_All | 1.3211 |
| at4g10380 | Unigene2151_All | -1.112 |
| at4g36220 | CL508.Contig1_All | 3.6815 |
| at5g52570 | CL10561.Contig1_All | -1.7671 |
| at4g35900 | CL1346.Contig4_All | -1.1287 |
| at4g26220 | CL2674.Contig1_All | 1.1927 |
| at5g18870 | Unigene8815_All | 2.6924 |
| at1g68380 | CL8811.Contig2_All | 1.4198 |
| at4g26540 | CL4702.Contig3_All | -1.5269 |
| at3g14370 | CL7613.Contig1_All | -1.5269 |
| at2g35880 | CL8109.Contig2_All | -1.3142 |
| at1g80870 | CL3609.Contig1_All | -1.3478 |
| at5g66820 | CL6144.Contig1_All | 2.8336 |
| at5g24270 | Unigene13533_All | 1.8411 |
| at1g70140 | Unigene4816_All | -1.7171 |
| at1g51680 | CL4859.Contig1_Al | 1.2945 |
| at2g44610 | CL7914.Contig2_All | 1.1074 |
| at3g13380 | CL4697.Contig1_All | 1.0021 |
| at2g36180 | CL928.Contig2_All | -1.1042 |
| at4g13390 | CL5079.Contig1_All | 2.0788 |
| at3g15760 | CL729.Contig3_All | 1.0225 |
| at4g25310 | CL3455.Contig2_All | 1.1831 |
| at2g18540 | Unigene20814_All | -1.9992 |
| at3g43630 | CL111.Contig3_All | -1.1628 |
| at4g35480 | CL4348.Contig2_All | -1.1629 |
| at5g04170 | Unigene17730_All | 3.4507 |
| at3g14820 | Unigene10860_All | 1.7809 |
| at3g49890 | Unigene12963_All | 1.1043 |
| at5g02890 | CL6865.Contig1_All | -1.35 |
| at4g13420 | CL10548.Contig1_All | 1.0304 |
| at5g24655 | CL5754.Contig2_All | 2.6817 |
| at3g09270 | Unigene7319_All | 2.6815 |
| at1g80980 | Unigene4240_All | -1.5707 |
| at4g36740 | CL4169.Contig2_All | -1.1988 |
| at5g57920 | CL3461.Contig3_All | 1.1612 |
| at5g15180 | CL8287.Contig1_All | 10.2896 |
| at4g33770 | Unigene6949_All | 1.5706 |
| at3g05470 | CL1926.Contig4_All | -1.2025 |
| at1g65450 | Unigene18436_All | -2.0732 |
| at1g72310 | Unigene14740_All | 1.3743 |
| at5g28080 | CL2889.Contig3_All | -2.1726 |
| at5g66800 | CL7810.Contig2_All | -2.1727 |
| at5g57550 | CL53.Contig1_All | -2.4517 |

**Table S7.** Gene analysis by MapMan in the root.

| **Gene ID (*Arabidopsis thaliana*)** | **Gene ID (*Salix matsudana*)** | **log2(root_Cd_FPKM/root_FPKM)** |
| --- | --- | --- |
| at2g24980 | CL1520.Contig1_All | 3.2683 |
| at2g18980 | CL4803.Contig3_All | -1.7086 |
| at1g30760 | CL1907.Contig5_All | 2.3547 |
| at5g60770 | Unigene12746_All | -3.1311 |
| at3g22460 | CL4768.Contig7_All | 2.0712 |
| at2g29500 | CL6892.Contig2_All | 1.3785 |
| at1g64660 | CL6894.Contig1_All | 1.1776 |
| at5g66390 | CL2717.Contig1_All | 1.4686 |
| at4g38970 | CL2715.Contig1_All | -1.2432 |
| at2g26380 | CL5735.Contig1_All | 1.4629 |
| at5g66920 | Unigene18884_All | -2.0139 |
| at2g22250 | CL2155.Contig1_All | 1.372 |
| at3g10040 | CL1616.Contig2_All | -3.3554 |
| atcg00080 | Unigene19218_All | -1.0238 |
| at2g01890 | Unigene19554_All | -1.0302 |
| at3g02550 | Unigene1104_All | -1.8155 |
| at5g24090 | CL4368.Contig1_All | 1.7274 |
| at1g75280 | CL8327.Contig1_All | 1.3509 |
| at2g47010 | Unigene18851_All | -1.0788 |
| at1g10670 | CL2362.Contig2_All | 1.2217 |
| at1g30700 | CL3504.Contig3_All | 1.3786 |
| at2g23910 | CL4422.Contig2_All | -1.0912 |
| at2g43590 | Unigene14498_All | 2.7931 |
| at2g21590 | CL4177.Contig6_All | -1.5196 |
| at1g14550 | CL7562.Contig1_All | 2.0273 |
| at3g46120 | Unigene4316_All | 1.068 |
| at1g76690 | CL3519.Contig3_All | 1.3906 |
| at5g22580 | Unigene3151_All | 1.5762 |
| at5g14780 | CL1166.Contig2_All | 1.0883 |
| at1g07820 | CL9334.Contig2_All | -1.8145 |
| at5g63850 | CL9629.Contig1_All | -1.6437 |
| at5g04970 | CL5786.Contig2_All | -1.4179 |
| at5g67400 | Unigene14856_All | 1.1967 |
| at5g13870 | CL5262.Contig4_All | -1.4556 |
| at4g37160 | Unigene11405_All | -1.3125 |
| at3g23800 | CL404.Contig2_All | 1.165 |
| at3g16150 | Unigene15161_All | -1.3494 |
| at1g73620 | CL2449.Contig1_All | -1.0052 |
| at5g44640 | CL1920.Contig4_All | 2.1273 |
| at2g36530 | CL9611.Contig2_All | -1.0636 |
| at2g30210 | CL7115.Contig1_All | -1.9207 |
| at2g06850 | CL5262.Contig1_All | -1.2105 |
| at1g73010 | Unigene6573_All | -1.3092 |
| at5g53550 | CL2482.Contig2_All | 1.1626 |
| at5g09800 | CL6253.Contig2_All | 1.3477 |
| at4g01830 | CL2946.Contig5_All | 1.5685 |
| at4g14130 | CL53.Contig5_All | -1.6778 |
| at1g60960 | Unigene8609_All | 1.0266 |
| at1g52400 | CL1920.Contig1_All | 2.3899 |
| at1g10020 | CL6292.Contig3_All | -1.4502 |
| at4g16600 | CL7225.Contig4_All | -1.0253 |
| at3g21420 | Unigene2003_All | -1.0365 |
| at4g37870 | CL2350.Contig1_All | -1.1096 |
| at5g14570 | Unigene19052_All | -1.2606 |
| at3g11180 | CL97.Contig1_All | 2.1701 |
| at5g46050 | CL8571.Contig1_All | 1.0442 |
| at4g08620 | CL4689.Contig6_All | 1.3188 |
| at3g51860 | CL1066.Contig4_All | 1.1492 |
| at3g23250 | Unigene590_All | 1.4312 |
| at5g54770 | CL4465.Contig1_All | -1.2292 |
| at3g53720 | CL5440.Contig2_All | 1.7636 |
| at5g59870 | Unigene11280_All | -1.0565 |
| at3g63140 | CL6319.Contig2_All | -1.1002 |
| at4g15930 | Unigene1107_All | -1.223 |
| at2g33710 | CL4274.Contig5_All | 1.5258 |
| at1g11545 | CL2311.Contig2_All | -1.1001 |
| at3g46230 | Unigene12934_All | 2.2532 |
| at3g48850 | Unigene4659_All | 3.0715 |
| at5g41560 | Unigene573_All | -1.0982 |
| at1g44830 | CL113.Contig2_All | -1.6815 |
| at5g47450 | CL7483.Contig1_All | 1.3156 |
| at3g23730 | CL53.Contig3_All | -1.2318 |
| at5g44330 | Unigene6322_All | -1.0279 |
| at5g45340 | CL1539.Contig3_All | -2.0411 |
| at1g76880 | CL8373.Contig3_All | -1.1388 |
| at5g15120 | CL7666.Contig1_All | -1.4286 |
| at3g09790 | CL2269.Contig5_All | 1.2011 |
| at2g30070 | Unigene12424_All | 1.4647 |
| at3g59850 | CL2649.Contig1_All | 1.3409 |
| at1g07400 | Unigene8500_All | 1.1904 |
| at4g32830 | CL464.Contig1_All | -1.1758 |
| at5g47060 | CL9882.Contig1_All | -1.312 |
| at1g78090 | Unigene19866_All | 1.1383 |
| at4g27410 | CL6120.Contig2_All | 1.192 |
| at4g00750 | CL8064.Contig3_All | -1.1028 |
| at3g63250 | CL6750.Contig2_All | 2.3493 |
| at3g09500 | Unigene6419_All | 1.3323 |
| at1g69700 | Unigene17794_All | -1.0767 |
| at3g43660 | CL111.Contig1_All | -1.2615 |
| at3g15500 | CL6120.Contig1_All | 2.0242 |
| at2g44520 | CL10611.Contig1_All | -1.2081 |
| at3g46030 | Unigene5154_All | -1.2297 |
| at1g20610 | Unigene16768_All | -1.0555 |
| at3g26060 | CL7947.Contig1_All | -1.2019 |
| at5g51460 | CL6722.Contig4_All | 1.3165 |
| at3g11050 | CL2210.Contig2_All | -1.1272 |
| at1g33760 | Unigene15054_All | -2.1322 |
| at4g23470 | Unigene19563_All | 1.0716 |
| at4g08950 | CL1447.Contig5_All | -1.4445 |
| at2g17230 | CL7277.Contig6_All | -1.6992 |
| at5g57530 | CL2071.Contig2_All | -2.7723 |
| at1g26820 | Unigene9179_All | -1.0257 |
| at3g27890 | CL6956.Contig1_All | -1.0342 |
| at5g59970 | Unigene10693_All | -1.1043 |
| at1g14700 | CL10272.Contig1_All | 1.6767 |
| at1g78000 | CL4689.Contig8_All | 1.8418 |
| at3g28490 | CL5512.Contig1_All | 1.0305 |
| at1g43860 | CL9227.Contig1_All | -1.2816 |
| at5g59910 | Unigene9231_All | -1.1283 |
| at3g66652 | Unigene8566_All | -1.7191 |
| at4g19230 | CL1539.Contig2_All | -1.7448 |
| at2g47580 | CL5800.Contig1_All | -1.0872 |
| at1g11170 | CL7190.Contig2_All | 1.1019 |
| at3g07360 | CL555.Contig2_All | 1.1962 |
| at4g13570 | CL574.Contig3_All | -1.1049 |
| at5g60820 | CL1653.Contig3_All | -1.0237 |
| at3g59660 | CL9939.Contig2_All | 1.2321 |
| at5g39180 | CL5623.Contig1_All | 1.3504 |
| at5g16600 | CL2248.Contig3_All | 1.1452 |
| at5g20410 | CL7381.Contig1_All | -1.0152 |
| at5g17160 | CL5151.Contig2_All | -1.195 |
| at2g02080 | CL10458.Contig3_All | -1.0765 |
| at1g79260 | CL7620.Contig1_All | 1.3272 |
| at5g24010 | CL8011.Contig3_All | -1.3698 |
| at3g20060 | CL9590.Contig1_All | -1.0287 |
| at1g69230 | CL4347.Contig1_All | -1.0546 |
| at5g26340 | CL6315.Contig1_All | 1.1897 |
| at1g77810 | CL5997.Contig2_All | 1.0683 |
| at5g63110 | Unigene4271_All | 2.4075 |
| at1g70290 | CL7703.Contig3_All | 1.0169 |
| at1g30230 | CL417.Contig2_All | -1.1646 |
| at5g62540 | CL7865.Contig1_All | 1.3567 |
| at5g44360 | Unigene14649_All | 1.1752 |
| at3g54420 | CL9594.Contig1_All | 1.9382 |
| at3g02280 | CL3755.Contig1_All | -3.0541 |
| at1g76760 | CL6505.Contig1_All | -1.5541 |
| at5g51380 | CL4659.Contig2_All | 1.1428 |
| at2g29590 | Unigene2505_All | -1.1776 |
| at4g23290 | CL78.Contig4_All | 1.0191 |
| at5g57480 | CL9225.Contig2_All | 1.6529 |

**Table S8.** Up-regulated genes in response to cadmium in the leaf.

| **Gene name** | **Gene ID** | **log2(leaf_Cd_FPKM/leaf_FPKM)** |
| --- | --- | --- |
| Hsps | CL1386.Contig3_All | 1.4433 |
|  | CL3410.Contig1_All | 1.6364 |
|  | Unigene19669_All | 4.7262 |
|  | Unigene14364_All | 1.0275 |
|  | Unigene16410_All | 1.3551 |
|  | CL8086.Contig2_All | 2.1225 |
|  | CL8086.Contig1_All | 2.8182 |
|  | CL3877.Contig2_All | 3.9162 |
|  | Unigene16409_All | 1.3368 |
|  | CL9190.Contig1_All | 2.542 |
|  | Unigene4756_All | 1.3495 |
|  | CL6966.Contig2_All | 1.2871 |
|  | CL10219.Contig2_All | 1.849 |
|  | CL1386.Contig2_All | 2.0743 |
|  | CL10219.Contig1_All | 2.8188 |
|  | Unigene16376_All | 4.0561 |
|  | CL1135.Contig2_All | 4.1639 |
|  | Unigene18849_All | 1.0048 |
|  | Unigene12934_All | 3.3093 |
|  | CL3697.Contig2_All | 1.1568 |
|  | CL8700.Contig3_All | 2.2567 |
|  | CL8430.Contig2_All | 2.454 |
|  | CL8430.Contig1_All | 2.4392 |
|  | Unigene23713_All | 4.5992 |
|  | Unigene4755_All | 1.3862 |
|  | Unigene12935_All | 2.2487 |
|  | Unigene18848_All | 1.1332 |
|  | Unigene7967_All | 1.9447 |
|  | CL1386.Contig1_All | 1.3894 |
|  | CL6892.Contig1_All | 12.9443 |
|  | Unigene12778_All | 1.1931 |
| GSH | CL2166.Contig1_All | 1.8363 |
| GST | Unigene9124_All | 1.4729 |
|  | Unigene10879_All | 1.0128 |
|  | CL8086.Contig2_All | 2.1225 |
|  | CL8086.Contig2_All | 2.1225 |
|  | CL8086.Contig1_All | 2.8182 |
|  | CL109.Contig2_All | 2.5288 |
|  | CL10219.Contig2_All | 1.849 |
|  | CL10219.Contig1_All | 2.8188 |
|  | Unigene15407_All | 3.1577 |
|  | CL541.Contig1_All | 1.7169 |
|  | CL9031.Contig1_All | 2.5277 |
|  | Unigene5044_All | 3.9566 |
|  | CL109.Contig1_All | 5.2842 |
|  | CL2625.Contig2_All | 1.973 |
|  | CL2625.Contig4_All | 4.4505 |
|  | CL10238.Contig1_All | 1.6467 |
|  | Unigene7319_All | 2.6815 |
| Sulfate transporter | CL2232.Contig1_All | 1.0923 |
|  | Unigene23053_All | 3.0312 |
|  | CL4689.Contig6_All | 1.2115 |
|  | CL4689.Contig5_All | 2.1398 |
|  | Unigene17138_All | 1.3915 |
|  | CL3689.Contig5_All | 1.6049 |
| ABC transporter | CL7396.Contig1_All | 3.1164 |
|  | CL7396.Contig2_All | 3.0738 |
|  | Unigene23379_All | 2.9268 |
|  | Unigene8466_All | 2.7427 |
|  | CL289.Contig1_All | 2.296 |
|  | CL720.Contig1_All | 2.2487 |
|  | CL5160.Contig1_All | 1.9909 |
|  | Unigene18500_All | 1.9624 |
|  | Unigene3406_All | 1.9268 |
|  | CL9883.Contig1_All | 1.8337 |
|  | CL4548.Contig2_All | 1.6519 |
|  | Unigene18501_All | 1.6304 |
|  | Unigene13614_All | 1.3282 |
|  | CL8462.Contig3_All | 1.2189 |
|  | CL5160.Contig2_All | 1.192 |
|  | CL11051.Contig1_All | 1.1511 |
|  | CL2946.Contig5_All | 1.0915 |
|  | Unigene19910_All | 1.0263 |
| ZIP transporter | CL3267.Contig3_All | 1.6945 |
| SOD | CL4365.Contig3_All | 2.0263 |
| POD | Unigene1102_All | 2.4014 |
|  | CL1919.Contig3_All | 1.7593 |
|  | CL7562.Contig1_All | 5.1937 |
|  | CL8265.Contig2_All | 2.0068 |
|  | Unigene19566_All | 1.2637 |
|  | CL4.Contig1_All | 1.8507 |
|  | CL4.Contig2_All | 2.4628 |
|  | CL1919.Contig1_All | 1.1586 |
|  | Unigene9130_All | 1.0743 |
|  | Unigene19174_All | 2.4673 |
|  | Unigene12772_All | 2.0737 |
|  | CL8186.Contig3_All | 1.4887 |
|  | CL8287.Contig1_All | 10.2896 |
| Glutathione reductase | CL2166.Contig1_All | 1.8363 |

**Table S9.** Up-regulated genes in response to cadmium in the root.

| **Gene name** | **Gene ID** | **log2(root_Cd_FPKM/root_FPKM)** |
| --- | --- | --- |
| HSPs | CL8086.Contig2_All | 1.4625 |
|  | CL3410.Contig1_All | 2.2045 |
|  | CL6892.Contig2_All | 1.3785 |
|  | CL8430.Contig2_All | 3.8906 |
|  | Unigene18849_All | 1.0552 |
|  | CL4837.Contig2_All | 1.2416 |
|  | CL8430.Contig1_All | 4.8632 |
|  | CL3877.Contig2_All | 5.2225 |
|  | CL1386.Contig2_All | 1.4411 |
|  | CL8700.Contig3_All | 1.4058 |
|  | Unigene19669_All | 3.8127 |
|  | Unigene18848_All | 1.0029 |
|  | Unigene7967_All | 1.5967 |
|  | CL4837.Contig1_All | 1.0476 |
|  | Unigene12934_All | 2.2532 |
|  | CL10219.Contig2_All | 1.2011 |
|  | Unigene8500_All | 1.1904 |
|  | Unigene16376_All | 3.2069 |
|  | Unigene5981_All | 1.611 |
|  | Unigene23713_All | 3.7057 |
|  | CL8820.Contig2_All | 2.1208 |
| Sulfate transporter | CL4689.Contig6_All | 1.3188 |
|  | CL4689.Contig8_All | 1.8418 |
|  | CL4689.Contig5_All | 1.1365 |
|  | Unigene23053_All | 1.3416 |
|  | CL4689.Contig3_All | 1.1709 |
|  | Unigene17138_All | 1.0199 |
| ABC transporter | CL2946.Contig5_All | 1.5685 |
|  | Unigene13614_All | 3.3838 |
|  | CL9883.Contig1_All | 3.8797 |
|  | CL6415.Contig1_All | 1.1396 |
|  | CL2946.Contig6_All | 1.0724 |
| ZIF transporter | Unigene8609_All | 1.0266 |
|  | CL2446.Contig2_All | 1.2754 |

**Table S10.** Genes used for cluster analysis.

| **Gene ontology term** | **Gene ID** |
| --- | --- |
| leaf-vs-leaf_Cd | |
| response to cadmium ion | Cl5174.Contig1_All, CL1386.Contig7_All, CL476.Contig21_All, CL2674.Contig1_All, CL1166.Contig2_All, CL6924.Contig1_All, CL7749.Contig2_All, CL3519.Contig1_All, CL8327.Contig2_All, CL1386.Contig3_All, CL3519.Contig2_All, Unigene14415_All, CL8893.Contig3_All, Unigene15407_All, Unigene10555_All, Unigene7967_All, CL1391.Contig2_All, Unigene5044_All, CL4932.Contig3_All, CL1281.Contig1_All, CL8832.Contig4_All, CL3519.Contig5_All, CL4768.Contig7_All, CL109.Contig2_All, CL47.Contig1_All, CL9190.Contig1_All, CL5562.Contig2_All, CL756.Contig2_All, Unigene15432_All, CL7749.Contig1_All, CL3610.Contig2_All, Unigene10173_All, CL6966.Contig2_All, CL4932.Contig2_All, CL1391.Contig5_All, CL1371.Contig6_All, Unigene19438_All, CL3519.Contig3_All, CL1454.Contig5_All, CL1386.Contig2_All, Unigene9097_All, CL1371.Contig5_All, CL7852.Contig2_All, CL1386.Contig4_All, CL109.Contig1_All, Unigene4739_All, Unigene16410_All, CL94.Contig1_All, CL2674.Contig3_All, Unigene16409_All, Unigene14946_All, CL1454.Contig6_All, CL8327.Contig1_All, CL5562.Contig3_All, CL9001.Contig2_All, Unigene15118_All, Unigene9124_All, CL1391.Contig6_All, CL1836.Contig2_All, Unigene17445_All, CL1386.Contig6_All, CL1281.Contig2_All, CL404.Contig2_All |
| defense response by callose deposition in cell wall | Unigene17821_All, Unigene17141_All, Unigene3262_All, CL2516.Contig1_All, Unigene19888_All, CL4548.Contig2_All, CL8017.Contig1_All, CL2516.Contig2_All, CL765.Contig3_All, CL8017.Contig2_All, Unigene4421_All |
| cell wall thickening | Unigene17821_All, Unigene17141_All, Unigene3262_All, CL2516.Contig1_All, Unigene19888_All, CL4548.Contig2_All, CL8017.Contig1_All, CL2516.Contig2_All, CL765.Contig3_All, CL8017.Contig2_All, Unigene4421_All |
| root-vs-root_Cd | |
| response to cadmium ion | CL8782.Contig1_All, Unigene19866_All, CL9156.Contig3_All, Unigene19438_All, CL9611.Contig2_All, CL9840.Contig3_All, CL3519.Contig3_All, CL1386.Contig2_All, CL1166.Contig2_All, Unigene20255_All, CL404.Contig1_All, CL7749.Contig2_All, CL9840.Contig1_All, CL3778.Contig3_All, Unigene26540_All, CL2715.Contig1_All, CL1475.Contig5_All, CL109.Contig1_All, CL417.Contig2_All, CL8327.Contig1_All, Unigene17795_All, CL2350.Contig1_All, Unigene7967_All, Unigene5226_All, Unigene17768_All, CL4768.Contig7_All, CL6256.Contig3_All, CL5562.Contig2_All, CL6956.Contig1_All, CL404.Contig2_All |
| defense response by callose deposition in cell wall | Unigene17821_All, CL2133.Contig4_All, CL1920.Contig4_All, Unigene17141_All, CL1920.Contig1_All, CL1920.Contig3_All, CL2516.Contig1_All, CL3778.Contig3_All, CL765.Contig3_All |
| cell wall thickening | Unigene17821_All, CL2133.Contig4_All, CL1920.Contig4_All, Unigene17141_All, CL1920.Contig1_All, CL1920.Contig3_All, CL9540.Contig6_All, CL2516.Contig1_All, CL3778.Contig3_All, CL765.Contig3_All |

**Table S11.** Down-regulated genes in response to cadmium in the leaf

| **Gene name** | **Gene ID** | **log2(leaf_Cd_FPKM/leaf_FPKM)** |
| --- | --- | --- |
| zinc finger family | Unigene12622_All | -12.1584 |
|  | CL3069.Contig5_All | -1.6371 |
|  | CL821.Contig2_All | -1.3433 |
|  | CL7815.Contig1_All | -1.3 |
|  | Unigene8431_All | -1.2107 |
|  | Unigene13347_All | -1.114 |
|  | CL9456.Contig2_All | -1.1074 |
| cellulose synthase gene | Unigene21630_All | -4.4657 |
|  | CL8346.Contig2_All | -1.6555 |
|  | CL8346.Contig2_All | -1.6555 |
|  | Unigene13064_All | -1.4505 |
| arabinogalactan protein | CL2728.Contig3_All | -4.465 |
|  | CL7127.Contig1_All | -3.6581 |
|  | CL2728.Contig1_All | -3.0326 |
|  | CL8736.Contig4_All | -1.8424 |
|  | CL8736.Contig2_All | -1.8397 |
|  | CL4622.Contig1_All | -1.6093 |
|  | CL2728.Contig2_All | -1.6051 |
|  | CL8736.Contig5_All | -1.5958 |
|  | CL4622.Contig5_All | -1.4432 |
| xyloglucan endotransglycosylase | CL53.Contig5_All | -4.2754 |
|  | Unigene21459_All | -4.1818 |
|  | CL53.Contig4_All | -3.4655 |
|  | CL53.Contig3_All | -2.8388 |
|  | CL9054.Contig2_All | -2.4882 |
|  | CL53.Contig1_All | -2.4517 |
|  | CL53.Contig2_All | -2.055 |
|  | CL5123.Contig2_All | -1.8646 |
|  | CL2311.Contig2_All | -1.8587 |
|  | CL5123.Contig1_All | -1.467 |
|  | CL3371.Contig2_All | -1.0194 |
| auxin-responsive protein IAA | CL2215.Contig3_All | -2.5512 |
|  | CL1370.Contig5_All | -2.5062 |
|  | CL1370.Contig3_All | -2.2738 |
|  | CL1370.Contig4_All | -2.22 |
|  | CL4299.Contig3_All | -1.8658 |
|  | CL7275.Contig2_All | -1.5829 |
|  | CL4299.Contig1_All | -1.5303 |
|  | Unigene15681_All | -1.3433 |
|  | CL10834.Contig2_All | -1.3348 |
|  | CL1370.Contig6_All | -1.3089 |
|  | CL10834.Contig1_All | -1.2483 |
|  | CL8893.Contig3_All | -1.2126 |
|  | CL2434.Contig1_All | -1.1945 |
|  | CL6796.Contig3_All | -1.0858 |
|  | CL2493.Contig2_All | -1.004 |

**Table S12.** Down-regulated genes in response to cadmium in the root.

| **Gene name** | **Gene ID** | **log2(root_Cd_FPKM/root_FPKM)** |
| --- | --- | --- |
| arabinogalactan protein | CL2728.Contig4_All | -1.9565 |
|  | CL3474.Contig3_All | -1.7066 |
|  | CL4622.Contig4_All | -1.4816 |
|  | CL4622.Contig5_All | -1.3979 |
|  | CL4622.Contig1_All | -1.1942 |
|  | CL4330.Contig3_All | -1.1447 |
|  | CL3584.Contig1_All | -1.0638 |
|  | CL4330.Contig2_All | -1.0042 |
| xyloglucan endotransglycosylase | Unigene10610_All | -1.7352 |
|  | CL53.Contig5_All | -1.6778 |
|  | CL53.Contig4_All | -1.5656 |
|  | CL5262.Contig4_All | -1.4556 |
|  | CL5262.Contig2_All | -1.3104 |
|  | CL53.Contig3_All | -1.2318 |
|  | CL2311.Contig2_All | -1.1001 |
|  | CL5262.Contig5_All | -1.0492 |
